# Automated Learning of a Dense Manifold of Electronic States and Electronic Energy Transfer and Reactions in Singlet O Collisions with N_2_

**DOI:** 10.34133/research.0992

**Published:** 2026-01-14

**Authors:** Qinghui Meng, Yinan Shu, Zoltan Varga, Dayou Zhang, Donald G. Truhlar

**Affiliations:** Department of Chemistry and Supercomputing Institute, University of Minnesota, Minneapolis, MN 55455-0431, USA.

## Abstract

Calculations of collisions involving excited electronic states play an important role in many high-energy environments, for example, in simulating thermal energy content and heat flux in flows around hypersonic reentry vehicles, and useful data are usually not available from either experiment or theory. We apply a deep learning framework—compatibilization by deep neural network—to automatically discover and fit a compatible potential energy matrix (CPEM) for singlet oxygen atom collisions with N_2_ in the ^1^*A′* manifold of N_2_O. The procedure yields not only a fit to the CPEM and its gradient but also analytic representations of the adiabatic potential energy surfaces and their gradients across a dense 13-state manifold; these potential energy surfaces are suitable for dynamics calculations of inelastic and reactive collisions across a broad range of collision energies extending above the dissociation threshold of N_2_. We propose a new asymptotically extended formulation of the curvature-driven coherent switches with decay of mixing (κCSDM) semiclassical dynamics method that resolves the conflict between differing symmetries of the interacting atom–diatom system and the completely separated final states. We use the new dynamics method with the analytic representation of potential surface gradients to compute electronically nonadiabatic cross-sections for N_2_(*X* ) + O(^1^S) collisions, primarily producing N_2_(*X*) + O(^1^D), N_2_(*A*) + O(^3^P), and NO(*X*) + N(^2^D). Our methods provide new capabilities for modeling electronic energy transfer under extreme conditions, with implications across chemistry, physics, and aerospace engineering.

## Introduction

Electronic energy transfer is a widely encountered process in atomic and molecular collisions. [1–3] The present article specifically treats collisions of atmospheric constituents under high-enthalpy conditions, but the methodology developed should be useful for a variety of applications in chemistry, physics, materials, and engineering.

Understanding collisions between nitrogen molecules and oxygen atoms is critical because of their role in atmospheric chemistry and understanding energy balance in the atmosphere [4,5]. Despite its importance, full modeling of atmospheric processes is limited by a sparsity of state-to-state rate constants for electronically, vibrationally, and rotationally inelastic interactions of atmospheric constituents. Of special interest is that these collisions often lead to the formation of electronically excited species and to the production of nitric oxide (NO), affecting—for example—the thermal and chemical environment encountered by hypersonic vehicles [6–9]. The detailed study of these nonadiabatic collisions can provide critical data for designing thermal protection systems and improving the efficiency of hypersonic flight as well as for modeling hypersonic vehicle reentry and modeling shock-tube experiments [10–28].

Simulating chemically reactive gas flows at high energies poses a formidable challenge due to the possibility of electronically nonadiabatic collisions, the simulation of which must consider multiple potential energy surfaces (PESs) and their couplings and must employ a dynamics method that accommodates electronic state changes and couplings. The required simulations involve 2 distinct stages. The first stage is the construction of an analytic representation of a consistent set of multiple global PESs, their gradients, and their state couplings. In the present work, the couplings will be calculated from the adiabatic PESs rather than from electronic wave functions. The second stage consists of dynamics simulations carried out using the surfaces, gradients, and couplings. Our recent work has been concerned with improved methodology for both stages [29–36], and the present work provides further improvements for both stages.

Molecular dynamics involving transitions between 2 or more electronic states is called electronically nonadiabatic dynamics or non-Born–Oppenheimer dynamics; it is intrinsically nonclassical because multiple electronic states generate multiple potential energy functions [37–40], whereas purely classical dynamics is always governed by single potential energy function. Dynamics calculations can be carried out in 2 ways: (a) by direct dynamics, which requires the potential surfaces, their gradients, and their couplings calculated on-demand by electronic structure calculations at every time step or (b) by using prefitted analytic potentials [41]. When the potentials and couplings are feasible to fit [42–45], the latter option is favored over direct dynamics because its lower cost (often lower by many orders of magnitude) enables a broader range of ensemble sampling, longer simulation times, and/or improved statistical accuracy for the convergence of a larger number of state-to-state transitions.

An important challenge in fitting coupled potential surfaces to an analytic representation is preservation of the correct topology. The most common way to carry out fits that preserve the topology is to transform from an adiabatic to a diabatic representation (diabatization) and fit the diabatic potential energy matrix (DPEM) [32,46,47]. Diabatization simplifies the process of fitting PESs because the elements of the DPEM are smooth functions of nuclear coordinates. In contrast, adiabatic PESs are not suitable for accurate fitting because they contain multidimensional cuspidal ridges, known as conical intersections, that correspond to electronic state degeneracies. The adiabatic PESs are not smooth at and near conical intersections, and the couplings in the adiabatic representation are singular vectors at these ridges, but the regions near the intersections are the most important ones for electronically nonadiabatic transitions [48,49], and so one should fit the surfaces and couplings well in these regions. This motivates the use of representations in which the surfaces and coupling are finite and smooth scalars. Typically, we restrict the dynamical treatment to a subspace of $N_{\text{states}}$ adiabatic states, whose best-estimate energies come from electronic structure calculations. Therefore, we seek a smooth matrix representation with the property that diagonalizing it yields the $N_{\text{states}}$ lowest adiabatic energies; this is called an adiabatic-equivalent basis.

While the process of transforming from an adiabatic to a smooth adiabatic-equivalent representation can be challenging, the reverse transformation to an adiabatic representation is straightforward, involving simply the diagonalization of a small matrix. Consequently, once the adiabatic-equivalent smooth matrix has been fitted, generating adiabatic surfaces at specific geometries required by the dynamics algorithm becomes an easy and inexpensive task.

Transforming to a diabatic representation can be onerous. We have developed machine-learning methods that use machine intelligence to simultaneously discover and fit an adiabatic-equivalent DPEM, which is tantamount to finding the diabatic transformation on-the-fly during the fitting process. This enables simultaneous diabatization and fitting for complicated systems with dense manifolds of states or in cases where electronic wave functions or nonadiabatic coupling (NAC) vectors are not available, i.e., for systems that are too challenging for traditional diabatization methods. Our successively improved diabatization methods include diabatization by deep neural network (DDNN) [31], permutationally restrained DDNN [50], and parametrically managed DDNN [34–36]. As compared to the original DDNN, the permutationally restrained DDNN method introduces restraints that promote invariance with respect to the permutation of identical atoms. The parametrically managed DDNN method additionally employs a low-dimensional potential and parametrically managed activation functions. As a result, the parametrically managed DDNN PESs can have greater accuracy in asymptotic regions where subsystems are distantly separated, which enables extrapolation of the neural network potentials into large asymptotic regions. (For triatomic systems, such as those treated in the present article, we define an asymptotic region as a region where an atom is far away from the remaining diatom or where all 3 atoms are separated from one another.) These methods create a DPEM using a set of target adiabatic PESs without relying on information about orbitals or couplings from the original electronic structure calculations. These DDNN methods have demonstrated good performance when applied to practical polyatomic chemical systems [31–36,51], especially for systems with dense manifolds of states [33,34,36] that present an almost an impossible task for traditional diabatization methods.

DDNN methods need to know the diabatic potentials at selected geometries on lower-dimensional grids. Therefore, DDNN methods do not completely eliminate the need to input some diabatic information; technically, the DDNN discovers the diabatic representation for the full range of geometries with the help of diabatic restraints at a small set of geometries. To eliminate that need, and in the spirit of learning coupled PESs in a fully automatic way, we have recently developed a new method called compatibilization by deep neural network (CDNN) [52]. Whereas the DDNN method obtains the adiabatic potential by diagonalization of a DPEM (the electronic Hamiltonian represented in a diabatic basis), the CDNN method obtains the adiabatic potential by diagonalization of a compatible potential energy matrix (CPEM, the electronic Hamiltonian represented in a compatible basis, which is implicit but not specifically generated).

To explain a compatible representation, we first review some concepts and explain the language we use because the language is sometimes used in different ways by different workers or in different contexts. We consider mixed classical–quantum methods in which nuclear motion is treated by classical trajectories representing narrow wave packets, and electrons are treated quantum mechanically by electronic wave functions $\psi_{J}\left(r;R\right)$ for each electronic state *J*, where **r** denotes electronic coordinates, and **R** denotes nuclear coordinates. In such methods, the electronic states are coupled by 2 kinds of terms: derivative couplings $\left\langle \psi_{I}\left|\frac{\partial }{\partial R}\right|\psi_{J}\right\rangle$ and electronic Hamiltonian couplings ${\left(H_{\text{elec}}\right)}_{\mathrm{IJ}}=\left\langle \psi_{I}\left|H_{\text{elec}}\right|\psi_{J}\right\rangle$. In an adiabatic representation, $H_{\text{elec}}$ is diagonal, and the derivative couplings are called NACs; the state coupling in the adiabatic representation is entirely due to the derivative couplings, which may be obtained as the gradients indicated above or as time derivatives or overlap integrals involving electronic wave functions at 2 times [30]. In a diabatic representation [32], the $\psi_{J}$ are smooth enough that the derivative couplings are negligible and the ${\left(H_{\text{elec}}\right)}_{\mathrm{IJ}}$ elements are smooth; the state coupling considered in a diabatic representation is entirely due to ${\left(H_{\text{elec}}\right)}_{\mathrm{IJ}}$ with *I* ≠ *J*. Furthermore, because the derivative couplings are negligible in a diabatic representation, the NACs can be obtained from the eigenvectors of the diagonalization of $H_{\text{elec}}$ [53]. In a compatible representation, the ${\left(H_{\text{elec}}\right)}_{\mathrm{IJ}}$ elements are smooth (in particular, they have continuous gradients even where the adiabatic potentials have cuspidal ridges). Furthermore, the implicit $\psi_{J}$ are assumed to be smooth enough that the derivative couplings are finite, and in a weak sense [54], this qualifies them to be called diabatic. Nevertheless, we do not call them diabatic because, when we do not use diabatic state functions or diabatic potential functions to guide the compatibilization, the $\psi_{J}$ are not necessarily so smooth that the derivative couplings are negligible. That is not a problem for us though because we will do the dynamics by the curvature-driven approximation [29,30,55]; the curvature-driven approach uses only the adiabatic PESs and their gradients; it does not need derivative couplings. We have demonstrated that the curvature-driven method gives good agreement with calculations based on derivative couplings for several problems, including the radiationless decay of ethylene [29], the photodissociation of ammonia [56], the ring openings of cyclohexahexadiene [57], γ-butyrolactone [58], and furanone [58], the photoisomerizations of *cis-* and *trans-*azomethane and *cis-*azobenzene [58], the stability of *trans*-azobenzene [58], and electronic excitation of O + O_2 _$\left({}^{3}\Sigma_{g}^{-}\right)$ → O + O_2_(^3^Δ_u_) [35]. Therefore, we only need to ensure that the adiabatic potentials are well fit; we do not need to ensure that good approximations to the NACs can be obtained from the eigenvectors of the CPEM. Note that in DDNN as well as CDNN, the $\psi_{J}$ are not actually calculated; they are implicit. We simply calculate ${\left(H_{\text{elec}}\right)}_{\mathrm{IJ}}$.

In the present article, we describe the procedure for an automatic learning of the 13-state ^1^*A*′ manifold of N_2_O using CDNN with parametric management in order to enforce correct behavior in asymptotic regions, and we use the resulting PESs and state couplings to carry out dynamics calculations of the electronically nonadiabatic quenching cross-sections of N_2_(*X* ^1^$\Sigma_{g}^{+}$) + O(^1^S) collisions, including the reactive production of NO. The dynamics calculations are carried out by a new extension of the coherent switches with decay of mixing (κCSDM) semiclassical dynamics method for non-Born–Oppenheimer processes. The new method is called asymptotically extended κCSDM, i.e., AE-κCSDM, and it resolves the conflict between differing symmetries of the interacting atom–diatom system and the completely separated final states, i.e., between the triatomic electronic symmetries and the diatomic electronic symmetries. This extension may be needed whenever the bimolecular products of an electronically nonadiabatic event have higher symmetry than the whole system. For example, in the present application, the collisions produce diatomic molecules with *C_∞v_* or *D_∞h_* symmetry, but the only global symmetry of the entire system is *C_s_*.

### The N_2_($r_{e}$) + O and NO($r_{e}$) + N asymptotes of the low-energy adiabatic states of N_2_O

Spin-orbit coupling is small for light atoms and is not included here. Therefore, the singlet, triplet, and higher-spin manifolds of N_2_O may be considered separately. Furthermore, we neglect Coriolis coupling (including the rotational coupling of the Renner–Teller effect). Then, since the geometries of a triatomic system have *C_s_* symmetry globally, states of *A*′ symmetry are uncoupled from those of *A*″ symmetry. As a result, we consider 8 uncoupled symmetry manifolds: ^1,3,5,7^*A*′ and ^1,3,5,7^*A*″.

Table 1 lists all possible combinations of low-lying energy states of N_2_ and atomic O up to the dissociation energy [*D_e_*(N_2_)] of the ground state of N_2_, which is 228.4 kcal/mol (9.90 eV) [59], and it shows the asymptotic atom–diatom states to which they are connected adiabatically. The table also contains the corresponding information for low-lying energy states of NO combined with atomic N up to the dissociation energy [*D_e_*(NO)] of the doublet ground state of NO, which is 152.6 kcal/mol (6.62 eV) [59,60]. The energies of N_2_ + O and NO + N are from the experimental literature [61,62]. The combinations in Tables 1 and 2 provide all the low-lying potential energy asymptotes of the adiabatic PESs of N_2_O. (For an alternative way of presenting the surface correlations, the reader is directed to the paper of Hopper [63].)

**Table 1.** The energy levels of N_2_ + O collision partners up to the dissociation energy of ground-state N_2_(*X*) and the energy levels of NO + N pairs up to the dissociation energy of ground state NO(*X*), and the number of potential energy surfaces in each symmetry manifold that are associated with each asymptote.^a^

Asymptote		*E*_dyn_ ^b^	Δ*V*_N2+O_^c^	Δ*V*_2N+O_^d^	Potential energy surfaces							
N_2_	O				^1^*A*′	^1^*A*″	^3^*A*′	^3^*A*″	^5^*A*′	^5^*A*″	^7^*A*′	^7^*A*″
*X* ^1^$\Sigma_{g}^{+}$	^3^P		0.00	−9.90			1	2				
*X* ^1^$\Sigma_{g}^{+}$	^1^D	0.00	1.97	−7.93	3	2						
*X* ^1^$\Sigma_{g}^{+}$	^1^S	2.22	4.19	−5.71	1							
*A* ^3^$\Sigma_{u}^{+}$	^3^P	4.26	6.22	−3.68	1	2	1	2	1	2		
*B* ^3^$\Pi_{g}$	^3^P	5.42	7.39	−2.51	3	3	3	3	3	3		
*W* ^3^$\Delta_{u}$	^3^P	5.45	7.42	−2.48	3	3	3	3	3	3		
*B′* ^3^$\Sigma_{u}^{-}$	^3^P	6.25	8.22	−1.68	2	1	2	1	2	1		
*A* ^3^$\Sigma_{u}^{+}$	^1^D		8.19	−1.71			3	2				
*a*′ ^1^$\Sigma_{u}^{-}$	^3^P		8.45	−1.45			2	1				
*a* ^1^$\Pi_{g}$	^3^P		8.59	−1.31			3	3				
*w* ^1^$\Delta_{u}$	^3^P		8.94	−0.96			3	3				
*X* ^1^$\Sigma_{g}^{+}$	^5^S		9.15	−0.75						1		
*B* ^3^$\Pi_{g}$	^1^D		9.36	−0.54			5	5				
*W* ^3^$\Delta_{u}$	^1^D		9.37	−0.53			5	5				
*A′* ^5^$\Sigma_{g}^{+}$	^3^P		9.77	−0.13			1	2	1	2	1	2
Number of surfaces					13	11	32	32	10	12	1	2
NO	N	Δ*V*_NO+N_^e^	Δ*V*_N2+O_^c^	Δ*V*_2N+O_^d^	^1^*A*′	^1^*A*″	^3^*A*′	^3^*A*″	^5^*A*′	^5^*A*″	^7^*A*′	^7^*A*″
*X* ^2^$\Pi_{r}$	^4^S	0	3.28	−6.62			1	1	1	1		
*X* ^2^$\Pi_{r}$	^2^D	2.38	5.66	−4.24	5	5	5	5				
*X* ^2^$\Pi_{r}$	^2^P	3.58	6.86	−3.04	3	3	3	3				
*a* ^4^$\Pi_{i}$	^4^S	4.77	8.05	−1.85	1	1	1	1	1	1	1	1
*A* ^2^$\Sigma_{g}^{+}$	^4^S	5.45	8.73	−1.17				1		1		
*B* ^2^$\Pi_{r}$	^4^S	5.69	8.97	−0.93			1	1	1	1		
*b* ^4^$\Sigma^{-}$	^4^S	6.04	9.32	−0.58	1		1		1		1	
*C* ^2^$\Pi_{r}$	^4^S	6.46	9.74	−0.16			1	1	1	1		
*D* ^2^$\Sigma_{g}^{+}$	^4^S	6.58	9.86	−0.04				1		1		
*B* ^2^$\Pi_{r}$	^2^D	8.08	11.36	1.46	5(3)	5(2)	5	5				
Number of surfaces					15(13)	14(11)	18	19	5	6	2	1

^a^ All energies are in eV with spin-orbit coupling removed, and vibrational energy is not included.

^b^ Energies relative to N_2_(*X*, *r_e_*) + O(^1^D).

^c^ Energies relative to N_2_(*X*, *r_e_*) + O(^3^P).

^d^ Energies relative to 2N(^4^S) + O(^3^P).

^e^ Energies relative to NO(*X*, *r*_*e*,NO_) + N(^4^S).

**Table 2.** Cross-sections (in Å^2^) as functions of initial conditions for N_2_(*X* ) + O(^1^S) collisions^a^

*E*_rel_	*v*	*j*	*E*_dyn_ ^b^	*σ*:1	*σ*:2	*σ*:3	*σ*:4	*σ*:5
				O(^1^D)	O(^3^P)	N(^2^D)	N(^2^P)	N(^4^S)
2	0	0	2.14	0.00	0.00	0.00	0.00	0.000
2.5	0	0	2.64	0.00	0.02	0.01	0.00	0.000
3	0	0	3.14	0.01	0.03	0.24	0.00	0.000
2	5	0	3.58	0.04	1.88	0.21	0.00	0.000
2	0	80	3.71	0.01	0.03	0.68	0.00	0.000
2.5	5	0	4.08	0.10	1.88	0.96	0.00	0.000
2.5	0	80	4.21	0.01	0.04	1.34	0.00	0.000
3	5	0	4.58	0.07	1.64	1.65	0.01	0.000
3	0	80	4.71	0.01	0.03	2.03	0.00	0.000
2	5	80	5.15	0.14	2.33	1.80	0.02	0.001
2.5	5	80	5.65	0.10	2.19	2.53	0.04	0.004
3	5	80	6.15	0.10	2.26	3.14	0.02	0.004

^a^ *v* is the initial vibrational quantum number of N_2_(*X*), *j* is the initial rotational quantum number of N_2_(*X*), *E*_rel_ is the initial relative translation energy (given in eV) of N_2_(*X*) + O(^1^D), and *σ* is a cross-section. The cross-section labels in the table include the states of the atom; the corresponding states of the molecules are specified in the first paragraph of the section below on Electronically nonadiabatic and reactive collision dynamics. Statistical errors are given in Table S9, and the number of trajectories leading to each outcome is given in Table S10. A scatter plot of cross-sections is given in Fig. S10.

^b^ *E*_dyn_ is dynamic energy (given in eV), which in this table is energy relative to N_2_(*X* $,r_{e}$) + O(^1^S), where $r_{e}$ denotes that the diatomic molecule is at rest at its equilibrium geometry. Dynamic energy is the sum of the initial relative-translational, vibrational, and rotational energies.

Table 1 shows that collisions of N_2_(*X*) with O(^1^S) begin only on the 4 ^1^*A*′ PES, and therefore, their cross-sections can be calculated using only the ^1^*A*′ manifold of states. (All the other low-lying asymptotes of N_2_ + O require at least 2 manifolds of states, and additional manifolds for collisions of other states can be calculated by the methods presented here.) The present article presents an efficient way to calculate a manifold of states by simultaneous icompatibilization and fitting with minimal human input for the diabatization subtask, and it illustrates the method by applying it to calculate the PESs of the ^1^*A*′ manifold and cross-sections for inelastic and reactive collisions of N_2_(*X*) with O(^1^S).

### PES learning

#### Learning strategy

One of our goals in learning analytic representations of PESs is that the sorted energy eigenvalues (from low to high) of our final CPEM should be close to the adiabatic potential energies from electronic structure calculations (which are introduced below). We aim to make the agreement especially good in asymptotic regions because the potential energies in these regions are used to assign initial state conditions and to analyze final states. Special attention to the asymptotic regions is critical not only for initial and final state definitions but also because a neural network potential has unpredictable behavior in regions where there are no data, but the asymptotic regions are infinite; thus, it is impossible to fill the space by the addition of more data.

To enforce the correct behavior of the analytic representation of the PESs in the asymptotic regions, we separate our final CPEM into 2 terms, namely, a pairwise additive (PA) term and a many-body term. Since we have only 3 atoms in the present case, the many-body term is a 3-body term and is called $\Omega^{3B}$, and since all 3-body interactions vanish in the asymptotic regions, the pairwise term controls the potential energies in those regions. To achieve an accurate PA term, we generate it by using reordered adiabatic diatomic potential curves, where the reordering depends on the diatomic internuclear distance. Although CPEMs are generally not diagonal, we enforce that there are no nonadiabatic transitions in asymptotic regions and therefore the $U^{\mathrm{PA}}$ term of the CPEM is diagonal.

Our final CPEM is written as

$$\Omega =f\left(\Omega^{3B}\left(R\right)\right)+U^{\mathrm{PA}}\left(R\right)$$ (1)

where $\Omega^{3B}$ is to be determined by a neural network, $f\left(\ldots \right)$ denotes a parametrically managed activation function, and the final predicted adiabatic potential energies are eigenvalues of Ω. The zero of energy is taken as 3 infinitely separated atoms. The role of the parametrically managed activation function in Eq. 1 is to decay its feed-in value (which is $\Omega^{3B}$) to zero in asymptotic regions, where the potential is given by the PA term $U^{\mathrm{PA}}$. In this way, the unpredictable behavior of the neural network potential in asymptotic regions becomes irrelevant.

Our fitting strategy involves the following steps:

A. We select a range of 3-body geometries to include in the fitting database, and we perform high-level electronic structure calculations for these geometries to form the database. We also perform electronic structure calculations on the diatomic potential energy curves (PECs).
B. We fit analytic functions to the diatomic PECs of NO and N_2_ from step A, and we use these to calculate the PA $U^{\mathrm{PA}}$ at all geometries of the 3-body database.
C. We employ a deep neural network of the parametrically managed CDNN type explained above, to fit $\Omega^{3B}$ such that the eigenvalues of the potential Ω of Eq. 1 provide a good fit to the 3-body potential energies of step A.

Subsections below and in Supplementary Materials discuss the ab initio calculations to provide the database and diatomic PECs, the computation of the PA potential, and the architecture of the parametrically managed CDNN and the learning process.

#### Step A—The learning database and electronic structure calculations

The first step in learning an analytic representation of coupled PESs is the creation of a database. In this work, the database comes from electronic structure calculations for ^1^*A*′ states with *C_s_* symmetry. We use Jacobi coordinates in which *r* and *d* are the N–N and N_2_–O distances, respectively, and *γ* is the angle between the N–N axis and the O–N_2_ vector. The coverage of nuclear configuration space includes geometries for which *r* and *d* vary in the range of 0.9 to 4.0 Å with an increment of 0.1 Å, while the angle *γ* ranges from 90° to 175° with an increment of 5° plus 179°. For Jacobi angles of 90°, 115°, and 135°, we also included geometries with smaller *d* (0.01, 0.05, 0.1, 0.2, and 0.8 Å) and with *r* ranging from 3.0 to 4.0 Å with an increment of 0.1 Å. For Jacobi angles of 90°, 135°, 160°, and 179°, we also included geometries with larger *d* (5, 6, 8, and 10 Å) and larger *r* (5, 6, 8, and 10 Å). This generates adiabatic energies at 18,337 geometries to be used for fitting.

Adiabatic potential energies for the 3-body database were calculated with extended multistate complete active space second-order perturbation theory (XMS-CASPT2) [64,65] based on state-averaged complete active space self-consistent field reference wave functions [66–70]. The active space consists of 12 electrons in 13 orbitals corresponding to all 2s and 2p orbitals of each atom with the removal of 2 doubly occupied orbitals and the addition of a 3s virtual orbital on oxygen. This yields 369,864 configuration state functions in *C_s_* symmetry. The state averaging used equal weighting of 13 states.

All electronic structure calculations were performed with the *OpenMolcas* program [71,72]. We used the cc-pVTZ basis set. In the XMS-CASPT2 calculations, we set the ionization potential–electron affinity shift to 0.35 a.u. and the imaginary shift to 0.1 a.u.

#### Step B—PA potential

The PA DPEM is written as

$$U^{\mathrm{PA}}=\sum_{i=1}^{3}U_{i}^{a–\mathrm{di}}\left(r_{i}\right)+E^{a}$$ (2)

where *i* is a diatom index, and $r_{1}$, $r_{2}$, and $r_{3}$ are the N1–N2, N1–O, and N2–O distances, respectively (where N1 and N2 are first and second N atoms), $E^{a}$ is a diagonal matrix with diagonal elements $E_{\delta }^{a}$, which is the sum of the 3 atomic energies when the system is completely dissociated in state δ, and $U_{i}^{a–\mathrm{di}}$ is the DPEM when diatom *i* is infinitely far from the other atom. Therefore, $U_{1}^{a–\mathrm{di}}\left(r_{1}\right)$ is the N_2_ + O DPEM with N_2_ distance equal to $r_{1}$, $U_{2}^{a–\mathrm{di}}\left(r_{2}\right)$ is the NO + N DPEM with NO distance equal to $r_{2}$, and $U_{3}^{a–\mathrm{di}}\left(r_{3}\right)$ is the NO + N DPEM with NO distance equal to $r_{3}$. For our system, $U_{2}^{a–\mathrm{di}}$ and $U_{3}^{a–\mathrm{di}}$ have the same analytic form because N2 is indistinguishable from N1.

All DPEMs are symmetric $N_{\text{states}}\times N_{\text{states}}$ matrices, with 1/2$N_{\text{states}}\left(N_{\text{states}}+1\right)$ unique nonzero matrix elements. Here, nonzero means that they are not constrained to be zero, although some matrix elements might happen to be zero at some geometries, and we require that in asymptotic-limit regions (which are regions with an atom infinitely separated from the diatom), the DPEMs are diagonal. This requirement means that both $U^{\mathrm{PA}}$ and $U_{i}^{a–\mathrm{di}}$ (with *i* = 1, 2, or 3) are diagonal (with diagonal elements $U_{\delta \delta }^{\mathrm{PA}}$ and ${\left[U_{i}^{a–\mathrm{di}}\left(r_{i}\right)\right]}_{\delta \delta }$, respectively), and it also means that in asymptotic-limit regions, the diabatic PESs and the adiabatic PESs are the same, except for the state ordering.

Define $V_{i}^{a–\mathrm{di}}$ as an adiabatic potential energy vector (a 13-dimensional vector) in which each element ${\left[V_{i}^{a–\mathrm{di}}\left(r_{i}\right)\right]}_{\alpha }$ is the adiabatic potential energy for asymptotic-limit atom–diatom type *i*. According to the previous paragraph, we have

$$\begin{array}{c} {\left[V_{i}^{a-\mathrm{di}}\left(r_{i}\right)\right]}_{\alpha }\equiv E_{\delta \left(\alpha ,i,r_{i}=\infty \right)}^{a}+{\left[U_{i}^{a-\mathrm{di}}\left(r_{i}\right)\right]}_{\delta \left(\alpha ,i,r_{i}\right)\delta \left(\alpha ,i,r_{i}\right)}\\ \;\text{in asymptotic regions} \end{array}$$ (3)

where $\delta \left(\alpha ,\;i,\;r_{i}\right)$ denotes the asymptotic state δ that corresponds to adiabatic state α in asymptotic-limit region *i* with the diatomic distance equal to $r_{i}$. The relation in Eq. 3 is called reordering of diabatic states, and the correspondence function $\delta \left(\alpha ,\;i,\;r_{i}\right)$ will be presented below. 

Based on the above, obtaining an analytic representation of $U^{\mathrm{PA}}$ requires the following steps:

1. Construct the potential curves for isolated diatomic molecules.
2. Assign the adiabatic and diabatic state numbers to determine the correspondence $\delta \left(\alpha ,\;i,\;r_{i}\right)$ in Eq. 3.
3. Fit the ${\left[U_{i}^{a–\mathrm{di}}\right]}_{\delta \delta }$ PEC for each state.

These steps are described in the Supplementary Materials.

**Fig. 1.**
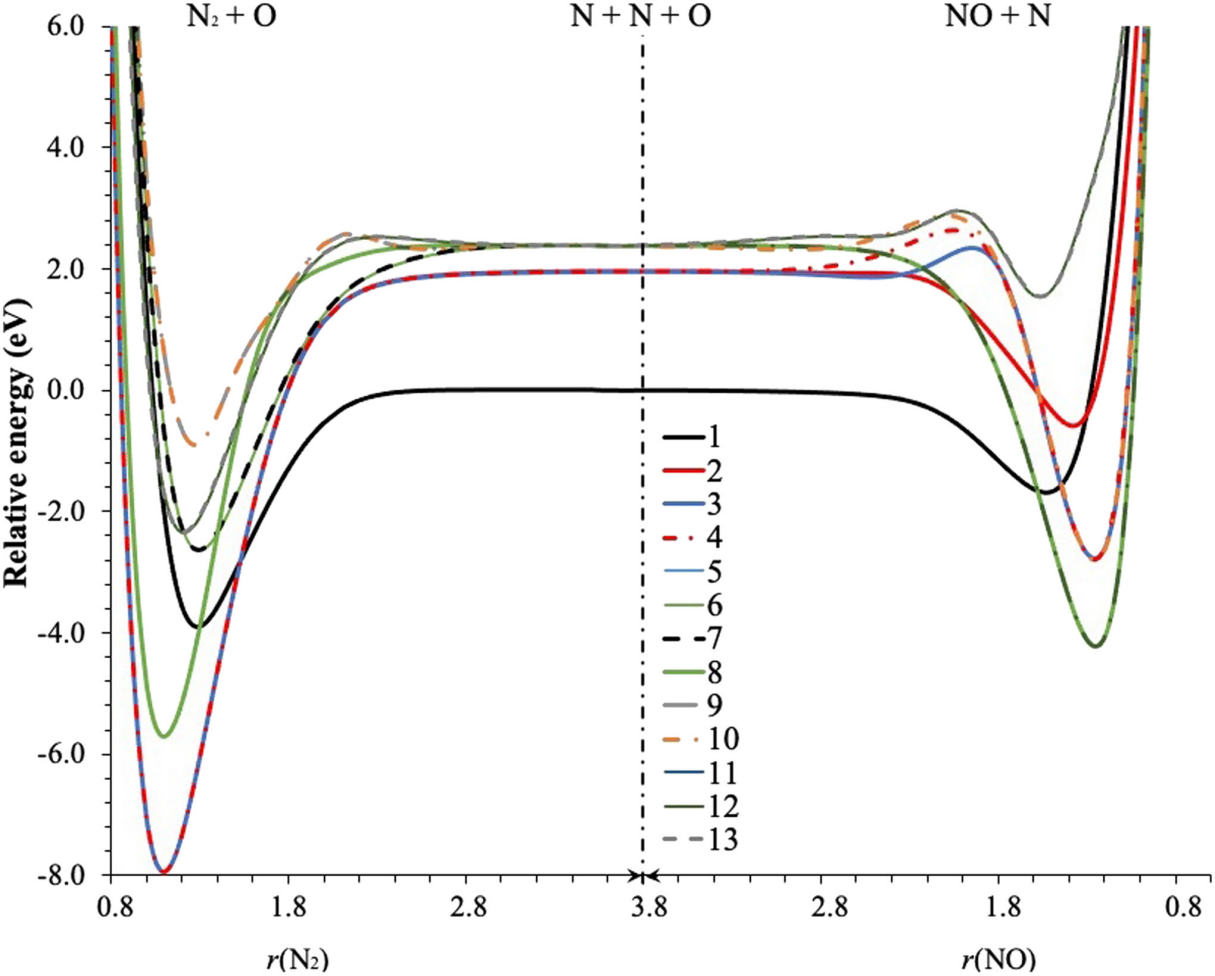
The $U_{\delta \delta }^{\mathrm{PA}}$ for N_2_ far from O (on the left) and NO far from N (on the right); the legend gives the values of δ. The center of the figure corresponds to complete dissociation. Starting in the center, the left side brings 2 N atoms together, and the right side brings the O toward one N.

The final pairwise potential is a function of the 3 internuclear distances and is defined for any triatomic geometry, either asymptotic or nonasymptotic. It is a diagonal matrix whose diagonal elements are given by inserting ${\left[U_{i}^{a–\mathrm{di}}\right]}_{\delta \delta }$ into Eq. 2. These diagonal elements are shown in Fig. 1, which shows that they cross, consistent with their designation as diabatic potentials.

#### Step C—Automatic learning with a parametrically managed CDNN

As discussed previously [31–35], and as illustrated schematically in Fig. S1, a neural network is used to obtain the CPEM. The CPEM is given by Eq. 1, where $U^{\mathrm{PA}}$ is diabatic, as explained above; however, no diabatic restraints are built into the first term on the right-hand side of Eq. 1, and therefore, the sum of the 2 terms is called a CPEM rather than a DPEM. Because we have now specified $U^{\mathrm{PA}}$, what remains is to specify *f* and to explain how we obtain $\Omega^{3B}\left(R\right)$. The neural network employs the parametrically managed CDNN method; Fig. S5 shows a schematic architecture of an *L*-layer parametrically managed CDNN.

The input layer for geometry *n* is $a_{n}^{1}$, the hidden layers are $a_{n}^{2}$ to $a_{n}^{L-2}$, the DPEM layer is $a_{n}^{L-1}$, and the adiabatic potential energy layer is $a_{n}^{L}$*.* Notice that $a_{n}^{1}$ is a 3-element vector where each element corresponds to one of the internuclear distances of N_2_O molecule, and the length of hidden layers are parameters that can be changed, which will be discussed below. The propagation from the input layer to hidden layers is carried out by

$$\begin{array}{c} a_{n}^{l+1}=g\left(z^{l}\right)=g\left[{\left(w^{l}\right)}^{T}a_{n}^{l}+b^{l}\right],\;l=1,2,..;L-3 \end{array}$$ (4)

where a superscript T denotes a transpose, $w^{l}$ and $b^{l}$ are respectively the connection weights and biases of layer *l*, and g is the hidden-layer activation function. The weight matrix $w^{l}$ is an $N_{l+1}\times N_{l}$ matrix, where $N_{l}$ is the length of vector $a_{n}^{l}$, and $N_{l+1}$ is the length of $a_{n}^{l+1}$; $b^{l}$ has length $N_{l+1}$. We use Gaussian Error Linear Unit (GELU) activation function [73] to prevent the vanishing gradient problem. The GELU activation function is defined as

$$g\left(z_{m}^{l}\right)=\frac{1}{2}z_{m}^{l}\left(1+\text{erf}\left(\frac{1}{\sqrt{2}}z_{m}^{l}\right)\right)$$ (5)

where erf is the error function, and the subscript *m* denotes the element *m* of vector $z^{l}$.

Propagation from the last hidden layer to the DPEM layer is done by

Ω~n3B=wL−2TanL−2+bnL−2 (6)

$$a_{n}^{L-1}=f\left({\tilde{\Omega }}_{n}^{3B};\;R_{n}\right)+{\tilde{U}}_{n}^{\mathrm{PA}}$$ (7)

where *n* denotes the geometry $R_{n}$ (which is a 3-element vector corresponding to the NN and 2 NO distances for geometry *n*), and ${\tilde{U}}_{n}^{\mathrm{PA}}$ and ${\tilde{\Omega }}_{n}^{3B}$ are like $U_{}^{\mathrm{PA}}$ and $\Omega_{}^{3B}$ in Eqs. 1 and 2 except that they are unique matrix elements of $U_{}^{\mathrm{PA}}$ and $\Omega_{}^{3B}$. Because we are considering 13 electronic states, $\Omega_{n}^{3B}$ and $U_{n}^{\mathrm{PA}}$ are 13 × 13 matrices (and $U_{n}^{\mathrm{PA}}$ is diagonal), whereas ${\tilde{U}}_{n}^{\mathrm{PA}}$ and ${\tilde{\Omega }}_{n}^{3B}$ are vectors with 91 elements $\left(\frac{N_{\text{states}}\left(N_{\text{states}}+1\right)}{2}=91\right)$. Similarly, $a_{n}^{L-1}$ is a 91-element vector, while $\Omega \left(R\right)$ in Eq. 1 is a 13 × 13 matrix.

Function $f\left(□\right)$ is the parametrically managed activation function whose role is to decay ${\tilde{\Omega }}_{n}^{3B}$ to zero when the geometry $R_{n}$ corresponds to an asymptotic region:

$$a_{n}^{L-1}\overset{R_{n}\to \text{asymptotic}}{\to }{\tilde{U}}_{n}^{\mathrm{PA}}$$ (8)

The parametrically managed activation function that we use to accomplish this is

$$f\left[z_{m};\;R_{n}\right]=f_{1}\left(r_{d,n}\right)f_{2}\left(r_{d,n}\right)z_{m}$$ (9)

where $r_{d,n}$ is a coordinate $r_{d}\;$(defined in the next paragraph) evaluated at geometry *n*, $z_{m}$ is element *m* of vector **z**, and

$$f_{1}\left(r_{d,n}\right)=0.5+0.5\;\text{tanh}\left(f_{f}\left(r_{d,n}-a_{f}\right)\right)$$ (10)

$$f_{2}\left(r_{d,n}\right)=0.5+0.5\;\text{tanh}\left(f_{f}\left(-r_{d,n}+b_{f}\right)\right)$$ (11)

where $f_{f}$, $a_{f}$, and $b_{f}$ are parameters with values of 1.2 Å^−1^, –2.0 Å, and 4.0 Å, respectively. The job of $f\left(□\right)$ is to decay all the elements of input vector *z* to zero if the nuclear configuration R is in a region where $f_{1}\left(r_{d,n}\right)f_{2}\left(r_{d,n}\right)$ is zero. The parameters *f*, *a*, and *b* and the choice of decay coordinate $r_{d,n}$ determine whether the nuclear configuration $R_{n}$ is the atom–diatom region. The separation parameter, $r_{d}$, for the parametrically managed activation function is taken as [51]:

$$r_{d}=\sqrt[{4}]{\sum_{i=1,j>i}^{3}{\left(r_{i}+r_{j}\right)}^{4}}-\sqrt[{4}]{\sum_{i=1}^{3}{r_{i}}^{4}}$$ (12)

The adiabatic potential energy layer is

$$a_{n}^{L}=\mathrm{diag}\left[M\left(a_{n}^{L-1}\right)\right]$$ (13)

where $M\left(a_{n}^{L-1}\right)$ denotes a conversion from the elements of the CPEM layer to a full CPEM (i.e., from the unique elements of the CPEM stored as a vector to the full matrix form), and $\mathrm{diag}\left[M\left(a_{n}^{L-1}\right)\right]$ denotes the diagonalization of the CPEM, and assign the eigenvalues into a vector $a_{n}^{L}-$ which is a 13-element vector.

The neural network weights and biases are optimized by minimizing a loss function that has 3 terms:

$$\begin{array}{c} C_{\mathrm{PM}-\text{CDNN}}=\frac{1}{2NN_{\text{states}}}\sum_{n=1}^{N}{\left|a_{n}^{L}-V_{n}\right|}^{2}\\ \;+\frac{\alpha_{2}}{2}\;\sum_{i=1}^{L-2}\;\sum_{p}\;\sum_{p^{\prime }}{\left|w_{{\mathrm{pp}}^{\prime }}^{l}\right|}^{2}+\frac{1}{2SN_{\text{states}}}\sum_{s=1}^{S}{\left|a_{s}^{L},-,V_{s}\right|}^{2} \end{array}$$ (14)

where $N_{\text{states}}$ is 13; the parameter $\alpha_{2}$ is set to 10^−4^; $w_{pp^{\prime }}^{l}$ is an element of the weight matrix between layers *l* and *l +* 1; and S equals $N_{p}N$, where $N_{p}$ is the number of nonidentity permutations of identical nuclei. The first term is one-half the mean squared error of the adiabatic potential energies at *N* geometries (here *N* = 18,337). The second term is a regularization designed to prevent overfitting, and the final term promotes the permutation symmetry of the optimized parametrically managed CDNN neural network by minimizing the error of adiabatic potential energies for geometries in the permuted adiabatic dataset. For N_2_O, $N_{p}=1$ because the permutation group of the 2 identical *N* atoms particles has 2 permutations (one of which is the identity).

The weights and biases were optimized with the L-BFGS algorithm [74], and the partial derivatives of CPEM elements with respect to weights and biases were obtained by automatic differentiation with *PyTorch*.

We have used all our data for training; we do not distinguish between training, testing, and validation sets. The regularization weight is set to 0.0001. We used the default convergence criteria implemented in PyTorch L-BFGS optimizer except that the maximum iteration was set to 20,000; our results show that convergence was reached in less than this maximum allowed number of iterations.

Once the CPEM Ω and its gradient with respect to the nuclear geometry are known, the adiabatic potential energy gradient with respect to the nuclear geometry can be computed [53],

∇RVα=∑δ,δ′NstatesTαδ∗Tαδ′∇RΩδδ′ (15)

where $\nabla_{R}$ denotes the derivative with respect to nuclear geometry, $V_{\alpha }$ is the adiabatic potential energy of adiabatic state α, $\Omega_{\delta \delta^{\prime }}$ is an element of final CPEM Ω, and $T_{\alpha \delta }$ is a matrix element of transforming matrix **T**, which diagonalizes Ω.

### Nonadiabatic collision dynamics

The nonadiabatic collision dynamics were calculated with the ANT 2025 software [75–77] using the curvature-driven coherent switching with the decay of mixing (κCSDM) method [29,30,37,78,79].

The CSDM and κCSDM algorithms are described elsewhere (CSDM [53,78]; κCSDM [29,30]), and here we give only a brief summary. CSDM is an extension of the semiclassical Ehrenfest (SE) method [80–82] to algorithmically model decoherence by means of decay of mixing. The SE method involves simultaneous propagation of the electronic density matrix and a nuclear-motion trajectory. In the present work, we carry out the dynamics in the electronically adiabatic representation. In this representation, SE trajectories are governed by a self-consistent potential calculated from the time-evolving electronic density matrix that adjusts coherently to nuclear motion by the time-dependent Schrödinger equation. In CSDM, the electronic density matrix also suffers an incoherent decay to an unmixed state according to an approximate Liouville–von Neumann equation [83]. The unmixed adiabatic state toward which the demixing occurs is called the pointer state [84], and the identity of the pointer state is switched as a function of time according to the fewest-switches [85] scheme. After the system leaves the 3-body region, the pointer state does not change, and the system emerges on the final pointer state.

In the present work, we changed the nonadiabatic force direction. The coherent nuclear electronic density matrix equation of motion is

P·C=−∑LρLL∇RVLdyn+∑LL′ReρL′LVLdyn−VL′dynσLL′κ,dynR··ΓLL′ΓLL′ (16)

where $V_{L}^{\mathrm{dyn}}$ is the effective dynamics potential energy of state *L*, which will be explained later this section, $\nabla_{R}V_{L}^{\mathrm{dyn}}$ is the effective dynamics potential energy gradient given below, $\rho_{LL^{\prime }}$ is the density matrix element between electronic states *L* and $L^{\prime }$, $\sigma_{LL^{\prime }}^{\kappa ,\mathrm{dyn}}$ is the effective curvature-driven time derivative coupling, which will also be explained below, $\overset{\cdot }{R}$ is the nuclear velocity, and $\Gamma_{LL^{\prime }}$ is the nonadiabatic force direction. In the present work, we used

$$\Gamma_{LL^{\prime }}=v_{\mathrm{vib}}$$ (17)

where $v_{\mathrm{vib}}$ is the vibrational velocity defined previously [30].

We will refer to asymptotic regions as regions where 2 of the internuclear distances are large enough that 2 of the 3 terms in Eq. 2 are negligible, and the asymptotic limit is when one atom is infinitely separated so only one term is nonzero. It is preferred to calculate curvature-driven dynamics using adiabatic PESs. However, for atom + diatom systems or molecule + diatom systems, there is a conflicting-symmetry problem when using a consistent adiabatic representation all along the trajectory. This is because the asymptotic-limit diatomics have higher symmetry (*D_∞h_* or *C_∞v_*) than the global symmetry (which is *C_s_* for atom–diatom collisions or *C*_1_ for diatom–diatom collisions). Here, we consider the ^1^*A*′ atom–diatom case. As discussed above, in the asymptotic limit, the PECs of different spatial and/or spin symmetry may cross, as one can observe in Fig. 1. However, when the atom is a finite distance away, even if that distance is large, the curves all have ^1^*A*′ symmetry and avoid crossing except on conical intersection seams, and the adiabatic representation always orders the PECs according to their energy. This conflicting symmetry problem also affects the dynamics, and the fix we employed in the fitting step is not sufficient for the dynamics. Because of this situation, we added an extra layer to the standard dynamics algorithm. The coupled-surface dynamics with this extra layer is called asymptotically extended dynamics

In asymptotically extended dynamics, when a trajectory is in an asymptotic region, the trajectory is propagated on asymptotic-limit potentials (i.e., on PECs of specific diatomic states) with zero couplings. This applies during initial state preparation, during trajectory pre-collision and post-collision propagation in asymptotic regions, and—crucially—during final-state analysis. For example, when a trajectory is propagating in an asymptotic region on a potential corresponding to N_2_(*X* ^1^$\Sigma_{g}^{+}$) + O(^1^D), vibrations of N_2_ and even dissociation of N_2_ should always be on a surface that corresponds to ${\left[U_{i}^{a–\mathrm{di}}\right]}_{\delta \delta }$ with δ equal to 2, 3, or 4, corresponding to one of the green blocks in Fig. S3A (the corresponding curves can be found in Fig. 1). Therefore, in such a situation, propagating on triatomic adiabatic surfaces is not proper. The asymptotically extended dynamics algorithm overcomes this conflicting-symmetry difficulty.

The trajectory in the region where all 3 atoms interact is treated in the adiabatic approximation, where adiabatic states do not cross except at conical intersections. However, initial and final states have higher symmetry and are classified as diabatic from the point of view of the triatomic system because the diatomic potentials cross. To handle this issue, we conduct the dynamics on effective adiabatic surfaces (called the dynamics surfaces) defined by

$$\begin{array}{c} \begin{array}{c} V_{\alpha }^{\mathrm{dyn}}=gV_{\alpha }+\left(1-g\right)\sum_{i=1}^{3}\left\{{\left[U_{i}^{a-\mathrm{di}}\left(r_{i}\right)\right]}_{\delta \left(\alpha ,i,r_{i,e}\right)\delta \left(\alpha ,i,r_{i,e}\right)}+h\left(i,r_{i}\right)E_{\delta \left(\alpha ,i,r_{i}=\infty \right)}^{a}\right\} \end{array} \end{array}$$ (18)

where $V_{\alpha }$ is true adiabatic surface α (i.e., the αth eigenvalue of matrix Ω defined in Eq. 1), the correspondences between the diabatic states of the $U_{\delta \delta }^{\mathrm{PA}}$ and the adiabatic states of $V_{\alpha }$ in each asymptotic region are in Table S1, $E_{\delta \left(\alpha ,i,r_{i}=\infty \right)}^{a}$ is defined in Eq. 2, ${\left[U_{i}^{a-\mathrm{di}}\left(r_{i}\right)\right]}_{\delta \left(\alpha ,i,r_{i,e}\right)\delta \left(\alpha ,i,r_{i,e}\right)}$ is the δth diagonal element of matrix $U_{i}^{a–\mathrm{di}}\left(r_{i}\right)$ , where $U_{i}^{a–\mathrm{di}}\left(r_{i}\right)$ (introduced in Eq. 2) is the diatomic PEC with the third atom placed infinitely far, *g* is a scalar indicator that signals whether the current geometry is in a nonasymptotic triatomic region or is in an asymptotic region, and *h* is a scalar indicator that signals which asymptotic region the system is in. Although *g* is a parameter of the asymptotically extended dynamics algorithm (not of the potential-surface-fitting algorithm), it is similar to the function *f* that is used in fitting the adiabatic surfaces; that is, it is close to zero in asymptotic regions and close to 1 in nonasymptotic regions, and it transforms continuously with continuous derivatives in passing between these 2 kinds of region.

The asymptotically extended dynamics algorithm ensures that the dynamics surface is the true fitted adiabatic surface $V_{\alpha }$ in interaction regions, where *g* is close to 1 and is equal to the dominant diatomic potential in addition to an energy of the third atom when *g* is close to 0. This can be seen most clearly by considering the following limiting cases of Eq. 18:

$$\begin{array}{c} \begin{array}{c} V_{\alpha }^{\mathrm{dyn}}=\left\{\begin{array}{cc} V_{\alpha } & g=1\\ {\left[U_{1}^{a-\mathrm{di}}\left(r_{1}\right)\right]}_{\delta \left(\alpha ,1,r_{1,e}\right)\delta \left(\alpha ,1,r_{1,e}\right)}+E_{\delta \left(\alpha ,1,r_{1}=\infty \right)}^{a} & g=0,r_{3}\gg r_{1},r_{2}\gg r_{1}\left(i.e.,\;N_{2}+O\right)\\ {\left[U_{2}^{a-\mathrm{di}}\left(r_{1}\right)\right]}_{\delta \left(\alpha ,2,r_{1,e}\right)\delta \left(\alpha ,2,r_{1,e}\right)}+E_{\delta \left(\alpha ,2,r_{2}=\infty \right)}^{a} & g=0,r_{3}\gg r_{2},r_{1}\gg r_{2}\left(i.e.,\;\mathrm{NO}+N\right) \end{array}\right. \end{array} \end{array}$$ (19)

where we set *r*_1_ as the N–N distance, and *r*_2_ and *r*_3_ are N–O distances.

Section S2 provides an example of applying the asymptotic extension, and it gives details of the *g* and *h* functions.

To remove spurious couplings in asymptotic regions [86], we make another change, namely, the curvature-driven time derivative coupling is diminished according to same function *g* that is used in Eq. 18:

$$\sigma_{LL^{\prime }}^{\kappa ,\mathrm{dyn}}={g\sigma }_{LL^{\prime }}^{\kappa }$$ (20)

where $\sigma_{LL^{\prime }}^{\kappa }$ is the standard [29,30] curvature-driven time derivative coupling.

The asymptotically extended dynamics algorithm is used for all dynamics calculations in the present article.

### Application details

#### Fitting the PESs

A key development in the present paper is that by switching from a diabatic basis to a compatible basis, we were able to avoid using diabatic restraints. We fit the 13 lowest ^1^*A*′ surfaces of N_2_O using adiabatic energies at 18,337 geometries. The initial weights and biases of the neural network were randomly generated and 200 runs with each random set were carried out for each network size to find the best fit. By experimenting with various network architectures, we found the best performance for a layer structure of (3, 405, 305, 205, 91). As explained above, the first number and last number are the size of the input vector and the number of unique CPEM matrix elements, respectively; these are specific to the ^1^*A*′ surfaces of N_2_O in the CDNN fitting. The 3 middle numbers are the numbers of neurons in the 3 hidden layers. The best-fitting results of various neural network sizes were compared in a previous study [35] that shows that the sizes (405, 305, 205) for the hidden layers are a good compromise of accuracy and efficiency. In order to run faster simulations, one more hidden layer was introduced, and the sizes of the hidden layers were reduced, resulting in a final neural network structure size of (3, 35, 65, 95, 125, 91). We made 200 runs with randomly generated initial weights to optimize the final network, and we accepted the result with the lowest mean unsigned error (MUE). As shown below, the MUE with the neural network architecture of (3, 35, 65, 95, 125, 91) is only 17% larger than that of the neural network architecture of (3, 405, 305, 205, 91).

#### Trajectory propagation

The equations of motion are integrated with the Bulirsch–Stoer integrator with the modification of Hack et al. [87]. Each trajectory begins with an atom–diatom separation of 10 Å. As discussed above, collisions of $N_{2}\left(X^{1}\Sigma_{g}^{+}\right)\;$ with OS1 start on the fourth ^1^*A*′ effective dynamics potential.

#### Cross-section calculations

We consider the following electronically inelastic processes:

N2X1Σg++OS1→N2X1Σg++OD1 (21)

N2X1Σg++OS1→N2A3Σu++OP3 (22)

We also calculated reactive cross-sections to form NO, as explained below. The following initial conditions are considered: 3 relative translational energies *E*_rel_ (2.0, 2.5, and 3.0 eV), 2 N_2_ vibrational quantum numbers *ν* (0 and 5), 2 N_2_ rotational quantum numbers *j* (0 and 80), and 4 ranges of impact parameters (0 to 1, 1 to 2, 2 to 3, and 3 to 4 Å, with each interval being denoted as $b_{L-1}\;\text{to}\;b_{L}$). For each of these sets of initial conditions, we ran 2,700 trajectories, where the initial orientations and vibrational phase are randomly sampled as explained elsewhere [75]. A small number of trajectories (about 0.5%) were discarded because they had not finished within 1 ps.

The electronically inelastic and reactive cross-sections are given by stratified sampling of impact parameters [75], which gives

σN2X+OS1→Y=π∑L=14bL2−bL−12PErelvjLY (23)

$$\langle P\left(E_{\mathrm{rel}},\;v,\;j,\;L;\;Y\right)\rangle =\frac{N_{\text{traj}}\left(E_{\mathrm{rel}},\;v,\;j,\;L;\;Y\right)}{N_{\text{traj}}\left(E_{\mathrm{rel}},\;v,\;j,\;L\right)}$$ (24)

where $N_{\text{traj}}\left(E_{\mathrm{rel}},\;v,\;j,\;L\right)$ is the total number of trajectories with impact parameter in the interval $b_{L-1}$ to $b_{L}$ and with initial conditions $E_{\mathrm{rel}},v,\text{and}\;j$, and $N_{\text{traj}}\left(E_{\mathrm{rel}},\;v,\;j,\;L;\;Y\right)$ is the number of those trajectories that end with condition Y. If desired, the cross-sections can be converted to nonthermal rate constants by multiplying by the relative velocity, which can be computed from the relative translational energy.

## Results and Discussion

### Potential energy surfaces

We obtained an MUE of 41 meV for the adiabatic energies for the (3, *405, 305, 205,* 91) network, and the MUE was increased to only 48 meV with the final (3, *35 ,65, 95, 125,* 91) network. Table S6 shows the MUEs of the final fit for 5 energy ranges; energies in this table are with respect to the energy zero at N(^4^S) + N(^4^S) + O(^3^P). With this zero of energy, Table 1 gives

• the energy of N2X1Σg+re+OS1 is –5.71 eV,
• the energy of N2X1Σg+re+OD1 is –7.93 eV,
• the energy of N2A3Σu+re+OP3 is –3.68 eV.

All of these are well below −2 eV, and Table S6 shows that the MUE for potential energies below −2 eV is only 34 meV. The MUE increases for higher energy ranges, primarily because it is harder to make quantitative fits on the steep repulsive walls, but that is acceptable because it is unnecessary for the fit potentials accurately in the repulsion regions that are not visited by trajectories. (Maximum absolute errors are in Table S13.)

**Fig. 2.**
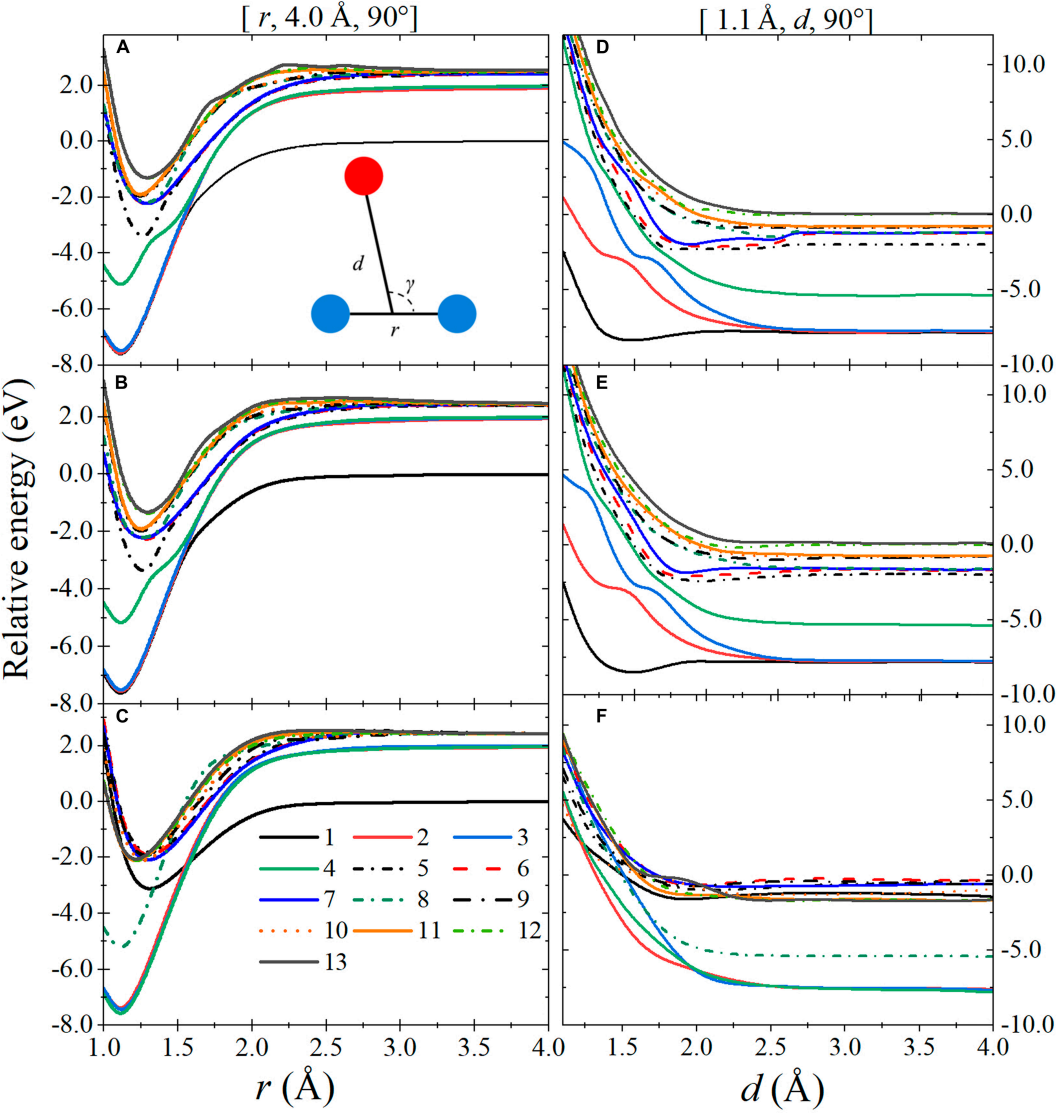
(A to C) Sample potential surface cut for geometries with *d* = 4 Å and *γ* = 90°. (D to F) Sample cut for geometries with *r* = 1.1 Å and *γ* = 90°. (A and D) Electronic structure calculations. (B and E) Fitted adiabatic energies. (C and F) Diagonal elements of compatibility matrix. The inset in panel (A) illustrates the Jacobi coordinates.

Electronic structure calculations and fitted adiabatic potential energies and diagonal elements of the compatible matrices are shown in Fig. 2 and Fig. S6 for 3 selected paths. We see that the fitted adiabatic energies show generally good agreement with the target adiabatic energies, and the diagonal elements of compatible matrices cross smoothly where the adiabatic energies show locally avoided crossings. Some of the curves for highly excited states are not completely smooth, but that is always expected when fitting a truncated manifold. However, the nonsmooth curves are at very high energy where they do not affect the presented dynamics.

An important feature of the ^1^*A*′ manifold is the collinear ground state of N_2_O. The geometry of the lowest-energy structure on the fitted ground-state PES of the N_2_O molecule was optimized with the CIOpt code [88]. The optimized N_2_O geometry has NN and NO bond lengths of 1.12 and 1.19 Å respectively, and the ONN angle is 180.0°. These coordinates may be compared to experimental values [89] of 1.12 Å, 1.19 Å, and 180° for NN and NO bond lengths and ONN angle. The computed frequencies at the optimized N_2_O minimum geometry are 665 and 665 for the degenerate bends, 1,161 for N–O stretching, and 2,063 cm^−1^ for N–N stretching; the experimental frequencies [89] are 589, 589, 1,285, and 2,224 cm^−1^. The computed dissociation energy for N_2_O → N_2_ + O(^1^D) is 3.68 eV, which may be compared to the experimental value of 3.49 eV (details of removing vibrational contributions from the experimental results so that they may be compared to the theoretical values are given in Table S7).

A comparison to the MCSCF/CI calculations of Hopper [63] is given in Section S3.

### Electronically nonadiabatic and reactive collision dynamics

In total, we calculated 12 × 4 × 2,700 = 129,600 trajectories. We calculated 5 kinds of cross-sections for N_2_(*X*) + O(^1^S) collisions:

1. Inelastic de-excitation of O to the ^1^D state: The final state is in arrangement N_2_ + O on adiabatic surfaces with *α* equal to 1, 2, and 3.
2. Inelastic excitation to N_2_(*A*) + O(^3^P): The final state is in arrangement N_2_ + O on the adiabatic surface with *α* equal to 5.
3. Reaction producing NO(*X*) + N(^2^D): The final state is in arrangement NO + N on adiabatic surfaces with *α* equal to 1, 2, 3, 4, and 5.
4. Reaction producing NO(*X*) + N(^2^P): The final state is in arrangement NO + N on adiabatic surfaces with *α* equal to 6, 7, and 8.
5. Reaction producing NO(*a*) + N(^4^S): The final state is in arrangement NO + N on the adiabatic surface with *α* equal to 9.

The electronically inelastic cross-sections and reactive cross-sections for N_2_(*X* ) + O(^1^S) collisions are given in Table 2. The reactive cross-sections for N_2_(*X* ) + O(^1^S) to produce NO are smaller than the electronically inelastic nonreactive cross-sections. Only one trajectory dissociated.

**Fig. 3.**
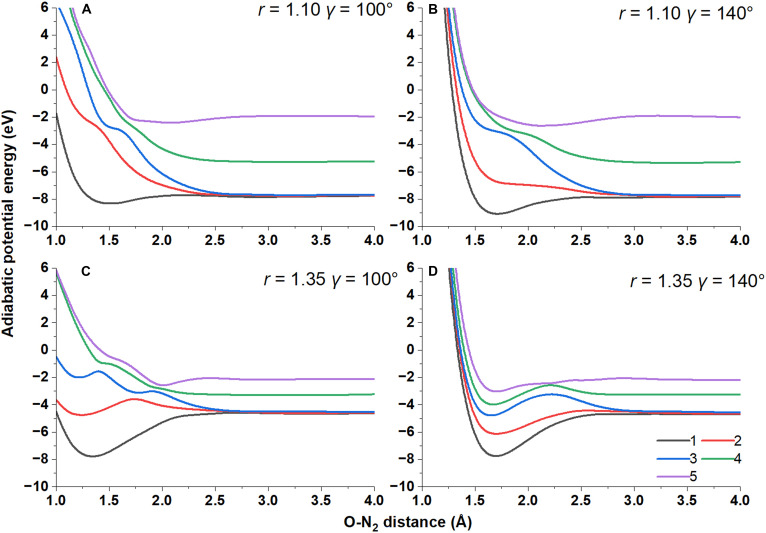
Adiabatic potential energy surface of the first 5 electronic states along the O to the center of mass of N_2_ distance. The 4 panels correspond to (A) *r* = 1.1 Å, *γ* = 100°; (B) *r* = 1.1 Å, *γ* = 140°; (C) *r* = 1.35 Å, *γ* = 100°; and (D) *r* = 1.35 Å, *γ* = 140°. The Jacobi coordinates are illustrated in the inset of Fig. 2.

Table 2 shows small cross-sections for electronically inelastic de-excitation transitions leading to N_2_(*X*) + O(^1^D) and larger cross-sections for N_2_(*A*) + O(^3^P) (processes 1 and 2, respectively). The reason for this is that the surface with *α =* 4 is well separated from those with *α =* 1, 2, 3. This is illustrated in Fig. 3 and Fig. S8, which show why inelastic excitation transitions (from the fourth to the fifth state) are more frequent than inelastic de-excitation transitions (from the fourth to the first, second, or third states) of O to the ^1^D state. This trend is discussed in more detail in Section S4.

Table 2 also shows that the initial vibrational energy of N_2_ is highly effective in promoting the electronically inelastic cross-sections. For example, the cross-section for N2X1Σg++OS1→N2A3Σu++OP3 is larger than or equal to 1.64 Å^2^ when the initial vibrational quantum number of N_2_ is 5, and is smaller than or equal to 0.03 Å^2^ when the initial vibrational quantum number of N_2_ is 0. This is explained by Fig. S8C and D and Fig. 3C and D, which show that the gaps are appreciably reduced when the N–N bond is stretched to 1.35 or 1.4 Å.

**Fig. 4.**
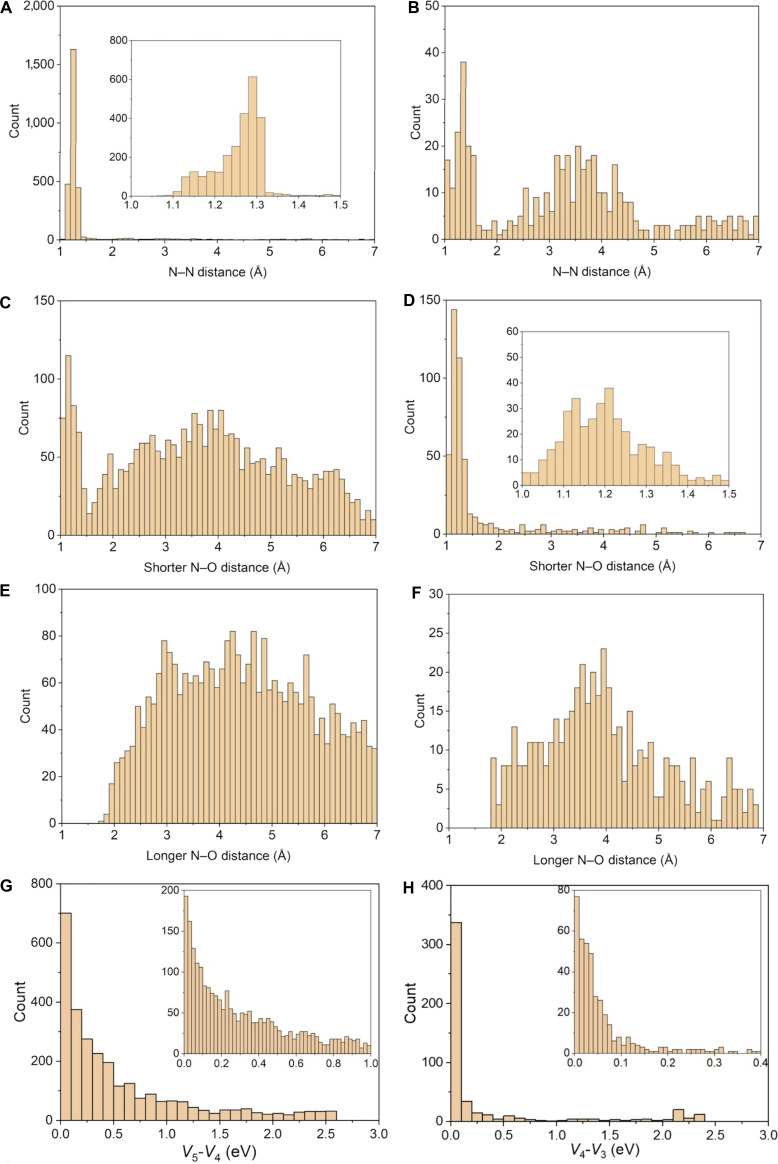
(A to F) Distribution of geometrical information at geometries where pointer state switches happen: (A, C, and E) for 4→5 pointer-state switch, (B, D, and F) for 3→4 pointer-state switch. (G and H) Distribution of potential energy gaps at geometries where a 4→5 pointer-state switch happens (G), and where a 4→3 pointer-state switch happens (H). The insets show finer details in the low-gap regions.

The numbers of 4→5 and 4→3 pointer-state switches are shown in Fig. 4. The majority of the pointer state switches are switches away from state 4, which is not surprising because the trajectories all start on the fourth electronic state. We find that 70% of the pointer-state switches are from *α* = 4 to *α* = 5, and 12% are from *α* = 4 to *α* = 3; this difference is consistent with the greater cross-sections for excitation than for de-excitation.

Figure 4 presents histograms illustrating the distribution of geometries (N–N distance, the shorter of the 2 N–O distances, and the longer of the 2 N–O distances) where pointer-state switches occur. Figure 4A, C, and E correspond to pointer-state switches from state 4 to state 5, and Fig. 4B, D, and F correspond to pointer-state switches from state 4 to state 3. One can observe from Fig. 4A that the majority of 4→5 pointer-state switches happen at short N–N distances within the range of 1.1 to 1.3 Å. In contrast, the majority of 4→3 pointer-state switches happen at short NO distances within the range of 1.0 to 1.5 Å, as can be seen in Fig. 4D. Figure 4 also shows the distribution of potential energy gaps where 4→5 and 4→3 pointer-state switches occur. Most of those switches happen when the gap is less than 0.4 eV.

Most of the chemical reactions that we observe are electronically adiabatic; in particular, 89% of the reactive collisions end up on adiabatic surface 4, which is the starting surface. Figure S9 shows the PES of the fourth electronic state as a function of the N–N distance and the smaller of the 2 N–O distances for the N–N–O angle equal to 90°, 120°, 150°, and 180°. Table 2 indicates that the N2X1Σg++OS1→NOX2Πr+ND2 reaction can occur at a dynamics energy as low as 2.64 eV—which corresponds to a potential energy of −3.13 eV relative to N(^4^S) + N(^4^S) + O(^3^P). Figure S9C and D show that the barrier to electronically adiabatic reaction can be as low as −3.0 to −4.0 eV (again, relative to 3 ground-state atoms), which is consistent with the dynamics.

## Conclusion

High-energy collisions involve dense manifolds of electronic states whose PESs and couplings are almost impossible to fit for practical simulations for electronically nonadiabatic dynamics. We previously circumvented this difficulty by using neural networks to discover diabatic or compatible representations of the potential energy matrix that are adiabatically equivalent. The compatible representation eliminates the requirement for diabatic data on reduced-dimensional cuts, and it is used here to fit coupled dense manifolds very conveniently. We showed that we could considerably decrease the size of the neural network architecture while suffering less than 10% increase in the error of energetic fitting as compared to our most accurate fits. This opens new opportunities for more affordable simulation of photochemistry and electronic energy transfer with high levels of electronic structure theory and minimal human guidance of the neural network, especially when combined with curvature-driven dynamics algorithms (such as κCSDM) that require only adiabatic PESs to treat electronically nonadiabatic dynamics.

Simulation of state-to-state dynamics in collisions involving atoms and diatomic molecules pose a special difficulty in that the symmetries of the initial and final states are higher than the symmetries maintained throughout the interaction. Here, we present asymptotically extended κCSDM to resolve this symmetry dilemma.

High-energy collisions of N_2_ with O play an important role in simulating thermal energy content and heat flux in flows around hypersonic vehicles and hence are very important for aerothermodynamic simulations of nonequilibrium and chemically reacting gas flows, especially for high-altitude simulations. Previous modeling of high-enthalpy gases had to use simple models for electronic energy transfer because data were not available either experimentally or theoretically. Progress in this field has been impeded by the lack of cross-sections for electronically nonadiabatic collisions, which are especially important for O atoms because of their relatively low-energy electronically excited states, and the lack of dynamics has been impeded by the unavailability of analytic representations of coupled PESs. Here, we combine the recent neural network compatible matrix method with the new asymptotically extended κCSDM to treat electronically nonadiabatic dynamics in the collisions O(^1^S) with N_2_ involving 13 coupled electronic states. We calculate cross-sections for excitation and de-excitation of O and for reaction to produce NO.

The techniques are broadly applicable, and they provide an opportunity for a new generation of aerothermodynamic simulations that include electronically inelastic cross-sections. To extend the framework to larger or more complex systems, a chief challenge will be the choice of global descriptors used to encode the molecular configurations and possibly other features that can guide the fitting of potentials [90,91] and their couplings [31]. Another challenge will be developing a more general way to interface asymptotic or fragment energetics with the supermolecule energies.

## Materials and Methods

A set of Fortran routines containing the fit is openly available in the current version of the CHEMical library of POTential energy surfaces in the PYthon (ChemPotPy) repository [92]. These routines can generate the potential surfaces, their gradients, and the couplings in both the diabatic and adiabatic representations. The Adiabatic and Nonadiabatic Trajectory (ANT 2025) software is available from Zenodo [77].

## Data Availability

The data are contained in the paper and the Supplementary Materials. There are no restrictions on the data.
