# Supplementary material for: Automated Learning of a Dense Manifold of Electronic States and Electronic Energy Transfer and Reactions in Singlet O Collisions with N2
Source: Research (Wash D C). 2026 Jan 14;9:0992. doi: 10.34133/research.0992 (PMC12799922; doi:10.34133/research.0992)
Supplement: Supplementary 1 — Figs. S1 to S10 Tables S1 to S13 [file research.0992.f1.zip › 251016_N2O_1Ap_SI.pdf]

SUPPLEMENTARY MATERIALS

Automated Learning of a Dense Manifold of Electronic States and Electronic Energy Transfer and Reactions in Singlet O Collisions with N_2

Qinghui Meng, Yinan Shu, Zoltan Varga, Dayou Zhang, and Donald G. Truhlar*

Department of Chemistry, Chemical Theory Center and Minnesota Supercomputing Institute, University of Minnesota, Minneapolis, Minnesota 55455-0431, USA

*email: truhlar@umn.edu

SI finalized on Sept. 6, 2025

TABLE OF CONTENTS

Section S1. The Pairwise Additive Potential S3

S1.1. Diatomic potential energy curves S3

S1.2. State numbering S4

S1.3. Accurate potential energy curves for the atom–diatom asymptotic regions S5

S1.4. The final pairwise-additive potential S7

Section S2. Asymptotically Extended Dynamics S8

S1.1. An example S8

S2.2. Details of *g* and *h* S9

Section S3. Comparison to Calculations of Hopper S9

Section S4. Additional Discussion of the Dynamics S10

Figure S1. Schematic diagram of learning strategy S12

Figure S2. Schematic illustration of diatomic potential energy curves S13

Figure S3. State connections between N_2 + O → N + N + O and NO + N → N + N + O S14

Figure S4. Schematic illustration of corrections to diatomic potential curves S15

Figure S5. Schematic illustration of the parametrically managed CDNN architecture S16

Figure S6. Sample potential surface cut for geometries with $d = 1$ Å and $\gamma = 90°$ S17

Figure S7. Values of *g* and three diatomic distances as functions of time S18

Figure S8. Adiabatic potential energy gaps S19

Figure S9. Contour plots of V_4 S20

Figure S10. Cross sections as functions of E_{dyn} for $N_2(X)$ + $O(^1S)$ collisions S21

Table S1. Relative energies (ΔE) and diabatic state order at asymptotic geometries S22

Table S2. Fitted parameters for the short-range term of ground-state N_2 S23

Table S3. Fitted parameters for V_n^{SR} in the asymptotic regions of N_2 + O S24

Table S4. Fitted parameters for the short-range term of ground state NO S25

Table S5. Fitted parameters for V_n^{SR} in the asymptotic regions of NO + N S26

Table S6. Mean unsigned errors (MUEs in meV) of the adiabatic energies S27

Table S7. Experimental dissociation energy of N_2O S28

Table S8. Adiabatic Potential Energies as function of the bond angle of NNO S29

Table S9. Statistical errors of cross sections as functions of initial conditions for $N_2(X)$+ $O(^1S)$ collisions S29

Table S10. Number of trajectories leading to each outcome as functions of initial conditions for $N_2(X)$+ $O(^1S)$ collisions S30

Table S11. Number of pointer-state switches for different pairs of states. S31

Table S12. The number of trajectories with 1, 2, and 3 pointer-state switches of each initial condition for $N_2(X)$ + $O(^1S)$ collisions S32

Table S13. Maximum errors in the final fit S33

Additional supporting information S34

Fortran routines S34

References S35

S1. The Pairwise Additive Potential

Figure S1 shows the strategy of separation and how employing a parametrically managed activation function is used to provide the correct physical behavior of our final analytic PES in asymptotic regions. The correct physical behavior in asymptotic regions is provided by the pairwise additive potential.

As input for developing the pairwise additive potential, we carried out XMS-CASPT2 calculations on the diatomic PECs. We did these calculations both with an atom far from the diatomic molecule and with the third atom not present. With the third atom not present, they are called $V_{\beta,i}^{\text{diatom}}$, where β denotes the electronic state and i denotes which diatomic pair is being calculated *(i* =1 for N_2 and i = 2 or 3 for NO). With the third atom present but far away, they are called $\left[\mathbf{V}_i^0(r_i)\right]_\alpha$, where α denotes the electronic state. Note that states of different diatomic symmetry cross when the third atom is not present, but they avoid crossing when it is present but far away, because then all states are $^1A'$. We did calculations for internuclear distances from 0.6 Å to 1.0 Å with a spacing of 0.05 Å, from 1.0 Å to 1.9 Å with a spacing of 0.01 Å, and from 1.9 Å to 4.0 Å with a spacing of 0.1 Å. For N_2, we used 10 active electrons in 10 active orbitals (all 2*s*, 2*p*, and 3*s* orbitals), and we state averaged the first 5 singlets and first 5 triplets. For NO, we used 11 active electrons in 10 active orbitals (all 2*s*, 2*p* and 3*s* orbitals), and we state averaged the first 5 doublets and first 5 quartets.

S1.1. Diatomic potential energy curves

This section considers the diatomic potential energy curves when the third atom is not present, i.e., the diatomic potentials in the asymptotic-limit region. For N_2, the required diatomic PECs in the 13-state $^1A'$ manifold of N_2O are those for the *X*, *A*, *B*, *W*, and *B′* states of N_2 and the *X*, *a*, *B*, and *b* states. of NO.

Because the XMS-CASPT2 diatomic dissociation energies do not agree well enough with experiment, we corrected the XMS-CASPT2 results by the scaled external correlation (SEC) [1] method:

$$V_{\beta,i}^{\text{diatom0}} = F_{\beta,i}\left(\left[\mathbf{V}_i^{\text{XMS-CASPT2}}(r_i)\right]_\beta - \left[\mathbf{V}_i^{\text{SA-CASSCF}}(r_i)\right]_\beta\right) + \left[\mathbf{V}_i^{\text{SA-CASSCF}}(r_i)\right]_\beta \qquad \text{(S1)}$$

$$V_{\beta,i}^{\text{diatom}} = V_{\beta,i}^{\text{diatom0}}(r_i) - V_{\beta=1,i}^{\text{diatom0}}(r_i = \infty) \qquad \text{(S2)}$$

where $V_{\beta,i}^{\text{diatom,0}}$ denotes our final computed SEC-corrected diatomic PEC for state β of diatom i with the third atom not present *(i* =1 for N_2 and i = 2 or 3 for NO); β can be one of the states of diatom described at the beginning of this paragraph; $\left[\mathbf{V}_i^{\text{SA-CASSCF}}\right]_\beta$ and $\left[\mathbf{V}_i^{\text{XMS-CASPT2}}\right]_\beta$ are the SA-CASSCF and XMS-CASPT2 energies; and $F_{\beta,i}$ is a SEC parameter chosen to improve the agreement with the experimental dissociation energy of the corresponding state.

Whereas we eventually need 13 states (thirteen values of adiabatic state index α for each i, we need only five values of β in eqs (S1) and (S2)for N_2 and only four values of β in eqs (S1)

and (S2) for NO. The final computed values of $F_{\beta,i}$ are 1.052, 1.164, 1.014, 1.107, 1.033 for the N_2 *X*, *A*, *B*, *W*, and *B′* states, respectively, and 0.974, 0.947, 0.982, 0.862 for NO *X*, *a*, *B*, and *b* states, respectively. With these $F_{\beta,i}$ values, the dissociation energies calculated by eq (S1) agree with the experimental values.

S1.2. State numbering

One may number the states in a degenerate manifold in any convenient way, and for the degenerate states of the diatoms, we have chosen a particular way and then used that way consistently. This subsection describes how we numbered both the degenerate and the nondegenerate states.

We obtained $\mathbf{V}_i^0$ by electronic structure calculations using a supermolecule (which is an atom-diatom complex) by putting one of the atoms far away, but not infinitely far. In this way, we obtain 13 adiabatic PECs for N_2 + O and 13 for NO + N. These are not the final $\mathbf{V}_i^{\text{a–di}}$, but this subsection explains how we obtain the final $\mathbf{V}_i^{\text{a–di}}$ by using the $\mathbf{V}_i^0$ to understand the characters of the states and to obtain the state numbering as functions of diatomic distances.

We start with the following preliminary approximation to $\left[\mathbf{U}_i^{\text{a–di}}(r_i)\right]_{\delta(\alpha,i,r_i)\delta(\alpha,i,r_i)}$:

$$\left[\mathbf{U}_i^0(r_i)\right]_{\delta(\alpha,i,r_i)\delta(\alpha,i,r_i)} = [\mathbf{V}_i^0(r_i)]_\alpha - E^{\text{a}}_{\delta(\alpha,i,r_i=\infty)} \quad \text{in asymptotic regions} \tag{S3}$$

We use this to obtain the $\delta(\alpha,i,r_i)$ function at each distance r_i. For most states and most r_i, $\delta(\alpha,i,r_i) \neq \alpha$ because, as functions of r_i, the $\left[\mathbf{V}_i^0(r_i)\right]_\alpha$ refer to the triatomic asymptotic region where the surfaces avoid crossing, whereas the $\left[\mathbf{U}_i^0(r_i)\right]_{\delta\delta}$ are defined for the asymptotic-limit region where the potentials are smoother and cross.

Panel a of Figure S2, which is schematic rather than showing our accurate curves, shows an avoided crossing in $V_{\beta,i}^{\text{diatom}}$, which occurs when the diatomic symmetries are the same. Panel b shows two $V_{\beta,i}^{\text{diatom}}$ that intersect as functions of the diatomic distance; this can occur if the two states have different diatomic symmetry. In contrast the $\left[\mathbf{V}_i^0(r_i)\right]_\alpha$ all look like panel a, and the $\left[\mathbf{U}_i^0(r_i)\right]_{\delta\delta}$ all look like panel c. In this example, we set the adiabatic ordering equal to the diabatic ordering at position C. Therefore, at position C, $\left[\mathbf{U}_i^0(\text{C})\right]_{11}$equals $\left[\mathbf{V}_i^0(\text{C})\right]_1$, and $\left[\mathbf{U}_i^0(\text{C})\right]_{22}$ equals $\left[\mathbf{V}_i^0(\text{C})\right]_2$. At position A, the ordering will change – which results in $\left[\mathbf{U}_i^0(\text{A})\right]_{22}$ equal to $\left[\mathbf{V}_i^0(\text{A})\right]_1$ and $\left[\mathbf{U}_i^0(\text{A})\right]_{11}$ equal to $\left[\mathbf{V}_i^0(\text{A})\right]_2$. At position A, we can see that $\delta(\alpha,i,r_i) \neq \alpha$; this is an example of state re-ordering.

We start by assigning the diabatic state ordering in the asymptotic-limit region to be the same as the adiabatic state ordering. Then, for each i, we decrease r_i along the grid specified above, and we switch the ordering of $\left[\mathbf{U}_i^0(r_i)\right]_{\delta\delta}$. every time we see an avoided crossing of the $\mathbf{V}_i^0$ curves, i.e., an avoided crossing of $\left[\mathbf{V}_i^0\right]_\alpha$ with either $\left[\mathbf{V}_i^0\right]_{\alpha+1}$ or $\left[\mathbf{V}_i^0\right]_{\alpha-1}$. The resulting diabatic state orderings at $N_2\left(r_{e,\text{NN}}\right)$ + O and $\text{NO}\left(r_{e,\text{NO}}\right)$ + N with ground-state equilibrium N_2 and NO distances are given in Table S1 (where $r_{e,\text{XY}}$ denotes the equilibrium internuclear distance of

diatomic molecule XY). The elements of the vector $\mathbf{E}^{\mathrm{a}}$ of Section 3.3 of the main text may be obtained by subtracting 5.71 eV from the second-last column of Table S1.

For example, the first row of Table S1 indicates that the first adiabatic electronic state of $^1A'$ N_2O at the $NO(r_{e,\mathrm{NO}})$ + N geometry has $NO(X^2\Pi_r)$ + $N(^2D)$ character and it corresponds to diabatic state 6, i.e., $[\mathbf{U}_2^0]_{66} = [\mathbf{V}_2^0]_1 - E_6^{\mathrm{a}}$, and $[\mathbf{U}_3^0]_{66} = [\mathbf{V}_3^0]_1 - E_6^{\mathrm{a}}$. A second example is that the first adiabatic electronic state of $^1A'$ N_2O at the $N_2(r_{e,\mathrm{NN}})$ + O geometry has $N_2(X^1\Sigma_g^+)$ + $O(^1D)$ character and it corresponds to diabatic state 4, i.e., $[\mathbf{U}_1^0]_{44} = [\mathbf{V}_1^0]_1 - E_4^{\mathrm{a}}$. See footnote a of Table S1 for a lengthier explanation.

Once the numbering is decided along the two types of diatomic internuclear distances, we connect the $[\mathbf{U}_i^0(r_i)]_{\delta\delta}$ points for each δ along the diatomic distance r_i, and this forms our preliminary atom-diatom diabatic PEC model, which is schematically shown in panel c of Figure S2 and which provides the left-hand side of eq (S3)

S1.3. Accurate potential energy curves for the atom–diatom asymptotic regions

Having determined the relation between α and δ at each distance r_i in each arrangement i, we now proceed to obtain $[\mathbf{U}_i^{\text{a–di}}]_{\delta\delta}$. There are two types:

Type 1. $[\mathbf{U}_i^0(r_i)]_{\delta\delta}$ has a consistent character along the diatomic distance. In such a situation, our final accurate $[\mathbf{U}_i^{\text{a–di}}]_{\delta\delta}$ for state δ can be equated to a diatomic curve $V_{\beta,i}^{\text{diatom}}$ plus an atomic excitation energy relative to the ground state of the third atom. For example,

$$[\mathbf{U}_{i=1}^{\text{a-di}}(r_{i=1})]_{\delta\delta} = \mathbf{V}_{\beta(\delta),i=1}^{\text{diatom}}(r_{i=1}) - E_\delta^{\mathrm{a}} \quad \text{(S4)}$$

where $\beta(\delta)$ denotes the diatomic state of N_2 that has the character associated with state δ, and ΔE_δ is the excitation energy of the O atom [relative to $O(^3P)$] for the atomic asymptote associated with state δ.

For N_2 + O, type 1 includes the following cases, where we give the character of the triatomic N_2 + O state followed by an arrow and the character of the 2N + O state:

1) $X^1\Sigma_g^+$ + $^1D \rightarrow {}^4S + {}^4S + {}^1D$, which corresponds to adiabatic states 1, 2, and 3 for N_2 + O and adiabatic states 2, 3, and 4 for N + N + O;
2) $A\,^3\Sigma_u^+$ + $^3P \rightarrow {}^4S + {}^4S + {}^3P$, which corresponds to adiabatic state 5 for N_2 + O and adiabatic state 1 for N + N + O;
3) $B\,^3\Pi_g$ + $^3P \rightarrow {}^4S + {}^2D + {}^3P$, which corresponds to adiabatic states 6, 7, and 8 for N_2 + O and adiabatic states 5, 6, and 7 for N + N + O;
4) $W^3\Delta_u$ + $^3P \rightarrow {}^4S + {}^2D + {}^3P$, which corresponds to adiabatic states 9, 10, and 11 for N_2 + O and adiabatic states 11, 12, and 13 for N + N + O.

Therefore, for N_2 + O → N + N + O, the only curves left are the curves that correspond to adiabatic states 4, 12, and 13 for triatomic N_2 + O and adiabatic states 8, 9, and 10 for N + N + O; these curves need to be described according to type 2. To make the state correspondences clear, panel a of Figure S3 shows the state connections with colored backgrounds.

For NO + N, type 1 includes the following cases:

1) $X^2\Pi_r$ + ^{2}D → ^{4}S + ^{2}D + ^{3}P (where we used the notation to denote the character of NO + N → N + N + O) which corresponds to adiabatic states 1 to 5 for NO + N, and adiabatic states 5 to 9 for N + N + O;
2) $a\,^4\Pi_i$ + ^{4}S → ^{4}S + ^{4}S + ^{3}P which corresponds to adiabatic state 9 for NO + N, and adiabatic state 1 for N + N + O; (3) $b\,^4\Sigma^-$ + ^{4}S → ^{4}S + ^{4}S + ^{1}D which corresponds to adiabatic state 10 for NO + N, and adiabatic state 2 for N + N + O.

The rest of the curves have complications of type 2. Panel b of Figure S3 shows the state connections with colored background for NO + N → N + N + O.

Type 2. $[\mathbf{U}_i^0(r_i)]_{\delta\delta}$ has a mixed character. And therefore, eq (S4)is only used near the equilibrium distance. For example, consider N_2 + O → N + N + O, the $X^1\Sigma_g^+$ + ^{1}S state of N_2 + O is the adiabatic state 4, however, dissociation of $N_2(X^1\Sigma_g^+)$ should lead to 2N(^{4}S), therefore, the complete-dissociation product should be N(^{4}S) + N(^{4}S) + O(^{1}S). However, this complete-dissociation product is not included in the 13 considered electronic state. And therefore, it has to be connected to a different complete-dissociation product. This is a complication in providing accurate PECs $[\mathbf{U}_i^{\text{a-di}}(r_i)]_{\delta\delta}$, and such a situation is explained in the next paragraph. In both panels a and b of Figure S3, the type-2 PECs are those without a colored background.

The correction scheme is schematically illustrated in Figure S4. In panel a of Figure S4, we distinguish three regions. The first region corresponds to the near-equilibrium region (which corresponds to N_2 + O or NO + N and which is labeled A on the abscissas of Figure S4), the second region (labeled B) is an intermediate region, and the third region (labeled C) is the complete-dissociation region (which corresponds to N + N + O). The preliminary diabatic PEC ($\mathbf{U}_i^0$, indicated as a black curve) obtained in subsection S1.2 may not be accurate enough. At the complete-dissociation limit, the accurate energy level is indicated by the red horizontal mark, and one can see that this accurate dissociation limit may come from a higher electronic state near the equilibrium region; this higher state is illustrated as red dashed curve. At the equilibrium region, the accurate energy level is indicated by the gray horizontal mark, and one can see that this accurate equilibrium well may come from an electronic state that dissociate to a different dissociation limit, this diatomic state is illustrated as gray dashed curve. This is illustrated by a specific example as follows. In our considered 13 electronic states, the 4th adiabatic state of N_2 + O is $N_2(X^1\Sigma_g^+)$ + O(^{1}S), which should lead to a complete-dissociation product of N(^{4}S) + N(^{4}S) + O(^{1}S) – which is not in the considered 13 electronic state, and therefore, the one of the N(^{4}S) + N(^{2}D) + O(^{3}P) complete-dissociation product, i.e. adiabatic state 8 of the complete-dissociation product, has to be connected with 4th adiabatic state of N_2 + O. And therefore, each region requires its own correction scheme. To obtain $[\mathbf{U}_i^{\text{a-di}}(r_i)]_{\delta\delta}$, we use different curves in different regions: in region A, we use eq (S4)and in region C, we use accurate atomic energies of the product relative to N(^{4}S) + N(^{4}S) + O(^{3}P). Such an accurate curve is schematically illustrated as the blue curve in panel b of Figure S4.

Notice that the reason for the existence of type-2 cases is that we seek adiabatic-equivalent DPEMs for the diatomics. Because the N_{states} lowest smooth diabatic states do not span the same space as the N_{states} lowest adiabatic states, we made some of the diabatic states disjointed

in order to obtain a set of N_{states} diabatic states that span the same space as the N_{states} lowest adiabatic states. This kind of treatment of diabatic states is always required in an adiabatic-equivalent treatment because the highest retained adiabatic states generally have interactions with the lowest omitted adiabatic states so, unless those interactions are only in inaccessible regions of space (which would be rare), it requires more than N_{states} diabatic states for an adiabatic equivalent diabatization of N_{states} adiabatic states.

At this point, we have obtained $\mathbf{U}_i^{\mathrm{a-di}}$ and $\mathbf{V}_i^{\mathrm{a-di}}$. In summary, for some of the diabatic states, namely those states that described as type 1 and having colored backgrounds in Figure S3, $[\mathbf{U}_i^{\mathrm{a-di}}]_{\delta\delta}$ is obtained by using accurate diatomic potential energy curves $V_{\beta,i}^{\mathrm{diatom}}$ in addition to an atomic energy; for the rest of the diabatic states, namely those states that have a white background in Figure S3, $[\mathbf{U}_i^{\mathrm{a-di}}]_{\delta\delta}$ has a disjointed character, and it is obtained by using different schemes in different regions.

S1.4. The final pairwise-additive potential

Each of the $[\mathbf{U}_i^{\mathrm{a-di}}(r_i)]_{\delta\delta}$ diabatic PECs is fitted by a specific functional form:

$$[\mathbf{U}_i^{\mathrm{a-di}}]_{\delta\delta} = W_{i,\delta}(r_i) \quad \text{(S5)}$$

where $W_{1,\delta}$ is the fitting function explained below. The fits for $\delta = 2, 3, 4$ for N_2 + O and $\delta = 5 - 9$ for NO + N are from previous work;[2,3] the other fits are new. Diabatic states 5 – 9 correlates with three $^1A'$ surfaces asymptotically connected to $N_2(X\,^1\Sigma_g^+)\ +\ O(^1D)$, as shown Figure S3, and to two $^1A''$ surfaces leading to asymptotically connected to $N_2(X\,^1\Sigma_g^+)\ +\ O(^1D)$.

For $i = 1$, which corresponds to N_2 + O:

$$W_{1,\delta}(r) \quad \text{(S6)}$$

$$= \begin{cases} \sum_{k=0}^{7} a_{k,\delta} \exp[-\alpha_\delta(\beta_\delta)^k r^2] & \delta = 1, 5\text{-}7, 11\text{-}13 \\ \left[\sum_{k=0}^{7} a_{k,\delta} \exp[-\alpha_\delta(\beta_\delta)^k r^2]\right] + \varepsilon_\delta \exp\left[-d_\delta(r - 1.7\text{Å})^2\right] & \delta = 8, 9, 10 \\ D_1^{\mathrm{SR,NN}}\left\{1 - \exp\left[-\sum_{k=0}^{6} a_k \left(\frac{r^4 - r_{e,NN}^4}{r^4 + r_{e,NN}^4}\right)^k (r - r_{e,\mathrm{N_2}})\right]\right\}^2 - D_1^{\mathrm{SR,NN}} + W_1^{\mathrm{D3(BJ)}}(r) & \delta = 2, 3, 4 \end{cases}$$

where $a_{k,\delta}$, a_k, α_δ, β_δ, and d_δ are coefficients to be fitted, $r_{e,\mathrm{N_2}} = 1.097$ Å, and $D_1^{\mathrm{SR,N_2}} = 224.9157$ kcal/mol. The fitting is achieved by optimizing the parameters and therefore at computed geometries (as described in section 3.2) the predicted value from $W_{1,\delta}(r_1)$ is as close as $[\mathbf{U}_1^{\mathrm{a-di}}(r_1)]_{\delta\delta}$. The fitted parameters for N_2 + O atom-diatom diabatic PEC model are given in Tables S2 and S3 in supporting information where Table S2 gives parameters for $\delta = 2, 3, 4$, and Table S3 gives parameters for the other δ.

For i = 2 or 3, which correspond to NO + N:

$$W_{2,\delta}(r) = \begin{cases} \sum_{k=0}^{7} a_{k,\delta}\, e^{-\alpha_\delta(\beta_\delta)^k (r)^2} & \delta = 1\text{-}4 \\ \left[\sum_{k=0}^{7} a_{k,\delta}\, e^{-\alpha_\delta(\beta_\delta)^k (r)^2}\right] + \varepsilon_\delta\, e^{-d_\delta\left(r-1.7\text{Å}\right)^2} & \delta = 10\text{-}13 \\ B_{\text{dNO}}\left(\sum_{k=1}^{10} a_k \left(\exp\left(-\frac{r-r_{e,\text{dNO}}}{\alpha_1} - \frac{\left(r-r_{e,\text{dNO}}\right)^2}{\alpha_2}\right)\right)^k\right) + W_2^{\text{D3(BJ)}}(r) & \delta = 5\text{-}9 \end{cases} \tag{S7}$$

where $a_{k,\delta}$, a_k, α_δ, β_δ, d_δ, α_1, α_2, and B_{dNO} are coefficients to be fitted, $r_{e,\text{dNO}} = 1.1508$ Å. The fitted parameters for N_2 + O atom-diatom diabatic PEC model is shown in Tables S4 and S5 in supporting information where the parameters for $\delta = 5\text{-}9$ is given in Table S4, and the rest states are given in Table S5.

The damped-dispersion term is[4]

$$V^{\text{D3(BJ)}}(r) = \sum_{k=6,8} \frac{S_k C_k}{r^k + \left(a_1\sqrt{\frac{C_6}{C_8}} + a_2\right)^k} \tag{S8}$$

where S_6 and S_8 are 1.0 and 2.0, respectively. The parameters a_1 and a_2 were set to 0.5299 a_0^2 and 2.2 a_0, respectively.[5] The constant C_6 is 19.7 a.u., and C_8 is calculated from C_6 for N-N interactions and for N-O interactions, the constant C_6 is 16.7 a.u., and C_8 is calculated from C_6.[6,7]

S2. Asymptotically Extended Dynamics

S1.1. An example

Using the example of section 4, assume the pointer state equals 4, i.e. trajectory is propagating on a self-consistent potential (SCP) that is dominated by dynamics surface 4, in the triatomic interaction region, we then have

$$V^{\text{SCP}} \approx V_4^{\text{dyn}}(g = 1) = V_4 \tag{S9}$$

Later, if an O atom is dissociated from the system, and if the pointer state does not change, this leads to

$$V^{\text{SCP}} \approx V_4^{\text{dyn}}(g = 0, r_3 \gg r_1, r_2 \gg r_1) = \left[\mathbf{U}_1^{\text{a–di}}(r_1)\right]_{88} + E_8^{\text{a}} \tag{S10}$$

Similarly, if an N atom is dissociated instead of an O atom is dissociated, the trajectory tends to propagate on a SCP that is,

$$V^{\text{SCP}} \approx V_4^{\text{dyn}}(g = 0, r_3 \gg r_2, r_1 \gg r_2) = \left[\mathbf{U}_2^{\text{a–di}}(r_1)\right]_{99} + E_9^{\text{a}} \tag{S11}$$

The gradient of the dynamics potential is

$$\nabla_{\mathbf{R}} V_\alpha^{\text{dyn}} = g\nabla_{\mathbf{R}} V_\alpha + (1-g)\nabla_{\mathbf{R}} U_{\alpha\alpha}^{\text{Asymp}} + \nabla_{\mathbf{R}} g\left(V_\alpha - U_{\alpha\alpha}^{\text{Asymp}}\right) \tag{S12}$$

where we have defined,

$$U_{\alpha\alpha}^{\text{Asymp}} = \sum_{i=1}^{3} \left\{ \left[\mathbf{U}_i^{\text{a-di}}(r_i) \right]_{\delta(\alpha,i,r_{i,e})\delta(\alpha,i,r_{i,e})} + h(i,r_i) E_{\delta(\alpha,i,r_i=\infty)}^{\text{a}} \right\} \tag{S13}$$

S2.2. Details of *g* and *h*

In the present work, for convenience, we use a form of the dynamics-potential damping function *g* that is very similar to the potential-fitting function *f* defined in Section 3.4:

$$g = g_1(r_{\text{d}}) g_2(r_{\text{d}}) \tag{S14}$$

where r_{d} is again taken as

$$r_{\text{d}} = \sqrt[4]{\sum_{i=1,j>i}^{3} (r_i + r_j)^4} - \sqrt[4]{\sum_{i=1}^{3} r_i^{\,4}} \tag{S15}$$

and where we use

$$g_1(r_{\text{d}}) = 0.5 + 0.5 \tanh\left(f_g (r_{\text{d}} - a_g) \right) \tag{S16}$$

$$g_2(r_{\text{d}}) = 0.5 + 0.5 \tanh\left(f_g (-r_{\text{d}} + b_g) \right) \tag{S17}$$

where f_g, a_g, and b_g are parameters with values of 1.2 Å^{-1}, –2.0 Å and 6.0 Å, respectively. Notice that the *g* function has its switch farther out into the asymptotic region than does the *f* function; this is required for good performance of the asymptotically extended dynamics.

For *h*, we used,

$$h(i, r_i) = \frac{e^{-r_i/\xi_i}}{\sum_{j=1}^{3} e^{-r_j/\xi_j}} \tag{S18}$$

where ξ_i is set to 1 Å for *i* = 1, 2, and 3.

S3. Comparison to Calculations of Hopper

It is possible to compare the present results to the MCSCF/CI calculations of Hopper[8] for the bending potentials of the lowest two states of NNO when the N–N distance is 1.128 Å, and the N–O distance is 1.184 Å. The comparison is shown in Table S8. The agreement is reasonably good except for the 2 $^1A'$ state at 110°. The MCSCF/CI gap at 110° is 4.0 eV, whereas the gap on our surface is only 0.6 eV. As a check we carried out a time-dependent density functional calculation of this gap with the MN15[9] exchange-correlation functional and the 6-311+G(d) basis set.[10] This gave an excitation energy of 0.63 eV, in excellent agreement with the present surface. The discrepancy may be due to some states being missed in the MCSCF/CI calculations. The

three lowest excitation energies at this geometry in the MN15 calculation are at 0.63 eV, 0.76 eV, and 4.05 eV. On the present fit, the three lowest states at 110° are 4.48, 5.09, and 8.37 eV, so the gap between the third state and the lowest state is 3.89 eV, which is close to the published MCSCF/CI gap between the second and first states.

S4. Additional Discussion of the Dynamics

Figure S7 shows the value of *g* and the three diatomic distances as functions of time for three prototypical trajectories where panels a and b correspond to first quenching process

$$\mathrm{N_2}\left(X^1\Sigma_\mathrm{g}^+\right) + \mathrm{O}(\,^1\mathrm{S}) \rightarrow \mathrm{N_2}\left(X^1\Sigma_\mathrm{g}^+\right) + \mathrm{O}(\,^1\mathrm{D}) \tag{S19}$$

panels c and d correspond to the electronic excitation process

$$\mathrm{N_2}\left(X^1\Sigma_\mathrm{g}^+\right) + \mathrm{O}(\,^1\mathrm{S}) \rightarrow \mathrm{N_2}(A^3\Sigma_\mathrm{u}^+) + \mathrm{O}(\,^3\mathrm{P}) \tag{S20}$$

and panels e and f correspond to a reactive trajectory of the form

$$\mathrm{N_2}\left(X^1\Sigma_\mathrm{g}^+\right) + \mathrm{O}(\,^1\mathrm{S}) \rightarrow \mathrm{NO}(X\,^2\Pi_\mathrm{r}) + \mathrm{N}(\,^2\mathrm{D}) \tag{S21}$$

The v, v', j, and j' are initial and final vibrational and rotational quantum numbers, and E_{rel} is the relative translational energy. We see that *g*, which is the indicator used in the asymptotically extended nonadiabatic dynamics algorithm, increases monotonically when the atom and diatom get close to each other and decreases monotonically when an atom recedes. This confirms the correct behavior of *g*.

The small cross sections for electronically inelastic collisions leading to $N_2(X)$ + $O(^1D)$ and $N_2(A)$ + $O(^3P)$ (processes 1 and 2 respectively) may be explained by consideration the separation of the surface with $\alpha = 4$ from those with $\alpha = 1$, 2, 3, and 5. This is illustrated in Figures 3 and S8. Figure S8 presents contour plots of the adiabatic potential energy gaps as functions of the O-atom position, with N_2O lying in the xy plane. The dark blue regions in Figure S7 show where the gaps between surfaces are small; these are region where nonadiabatic transitions are most likely. Panels a and c correspond to the gap between α = 4 and α = 3, while panels b and d correspond to the gap between α = 5 and α = 4. For panels a and b, the N–N bond length is fixed at 1.1 Å with nitrogen atoms at (x, y) = (0, ±0.55 Å), whereas for panels c and d the bond length is 1.4 Å with nitrogen atoms at (0, ±0.70 Å). We see that both the adiabatic potential energy gap between $\alpha = 4$ and $\alpha = 3$ and the adiabatic potential gap panel between $\alpha = 5$ and $\alpha = 4$ are reasonable large for most accessible geometries.

The greater magnitude of inelastic excitation transitions (from the 4th to the 5th state) as compared to inelastic de-excitation transitions (from the 4th to the 1st, 2nd, or 3rd states) of O to the ^{1}D state is also supported by Figures 3 and S8, which reveal that the energy gaps between surfaces 4 and 5 are generally smaller than those between surfaces 4 and 3. When the N–N bond length is 1.1 Å, the gap between α = 4 and α = 3 is larger than that between α = 5 and α = 4 in the region $x < 2.0$ Å and $y < 2.5$ Å, as shown in panels a and b of Figure S8. A similar trend is observed for the 1.4 Å bond length in panels c and d. Smaller energy gaps promote more frequent pointer-state switches, which explains why transitions from 4 to 5 dominate. A detailed analysis of pointer state switching is given below.

The total number of pointer state switches for each pair of electronic states is shown in Table S11. We see that the majority of the pointer state switches are switches away from state 4, which is not surprising because the trajectories all start. on the 4th electronic state. We find that 70% of the pointer-state switches are from $\alpha = 4$ to $\alpha = 5$, and 12% are from $\alpha = 4$ to $\alpha = 3$; this difference is consistent with the greater cross sections for excitation than for de-excitation.

In total, we calculated $12 \times 4 \times 2700 = 129{,}600$ trajectories. Of these, there are a total of 87 trajectories with two switches of the pointer state switches and five trajectories with three. The rest of the trajectories have zero or one switch. Thus, despite the dense manifold of states, the great majority of the trajectories have at most one pointer state switch. The number of pointer-state switches for different pairs of states is given in Table S11. The number of trajectories with 1, 2, and 3 pointer-state switches for each initial condition is shown in Table S12. That table shows that there are a found a grand total of 4,222 pointer-state switches in the trajectories.

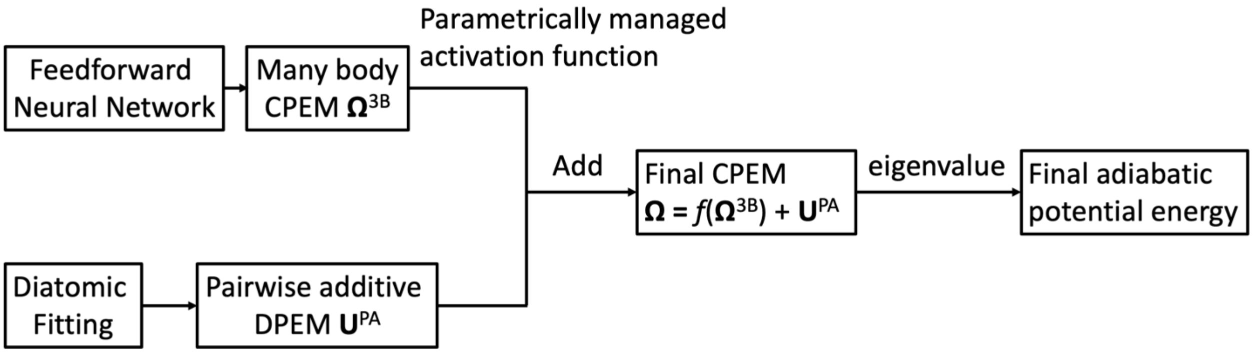

Figure S1. Schematic diagram of learning strategy.

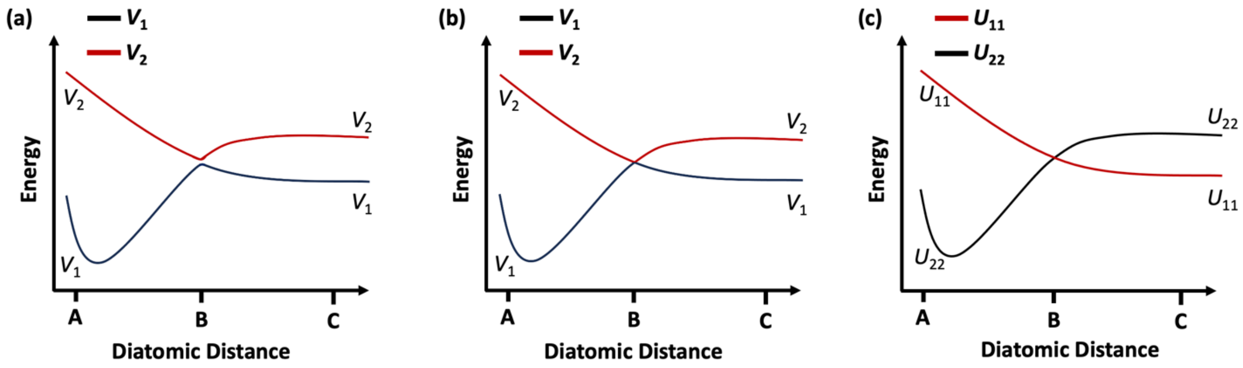

Figure S2. Schematic illustration. (a) Adiabatic PECs that avoid crossing. (b) diabatic PECs that cross. (c) diabatic PECs.

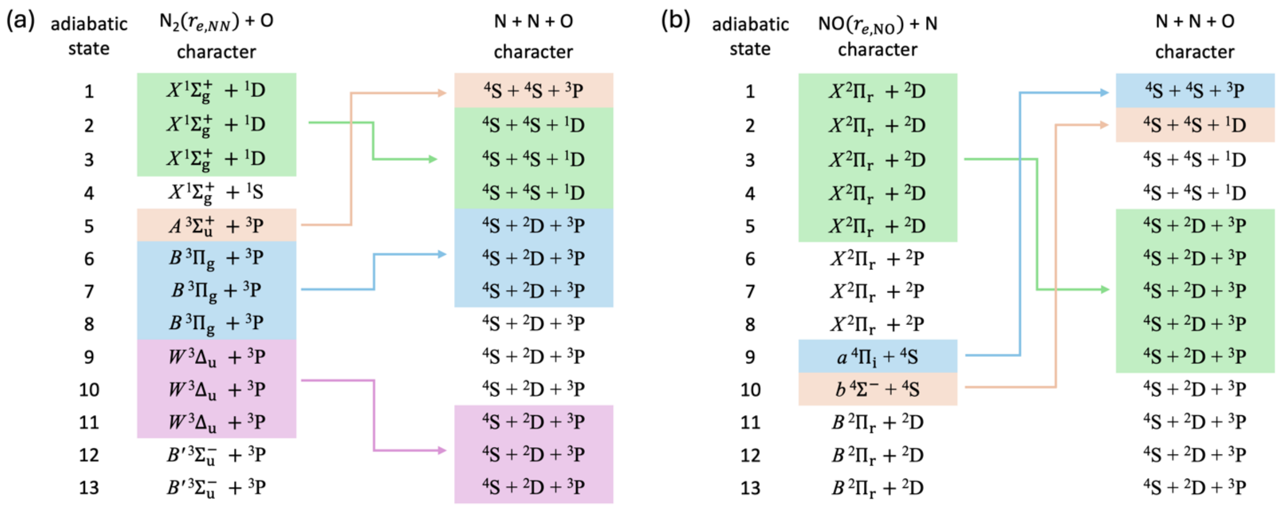

Figure S3. State connections between N_2 + O → N + N + O and NO + N → N + N + O. Colored entries are type 1, that, they are cases where the diatomic curve is involved in the 13 electronic states for the whole range of diatomic distances.

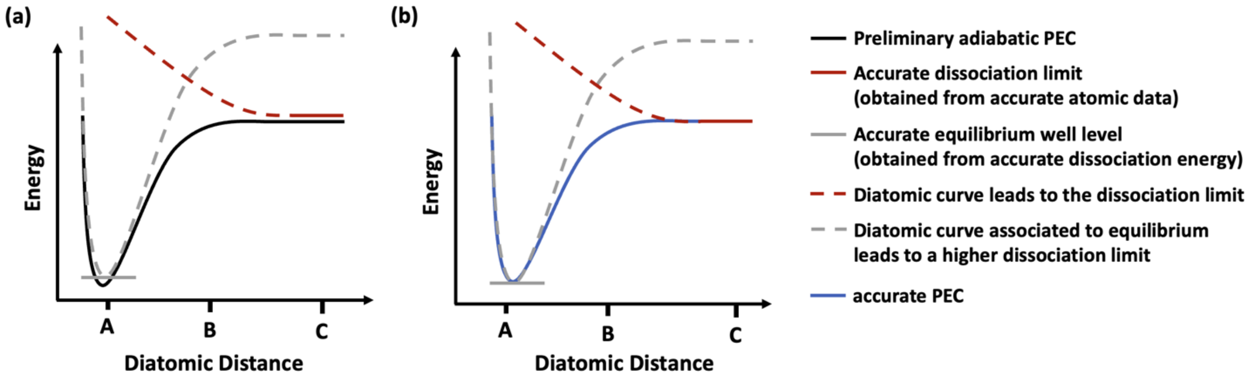

Figure S4. Schematic illustration of corrections to diatomic PECs. (a) The preliminary diatomic PEC $\mathbf{V}_i^0$ from supermolecule calculations is shown as a black curve, and two of the diatomic curves calculated with a third atom present ($V_{\beta,i}^{\mathrm{diatom}}$ (with two values of β and the same *i)* plus an atomic energy) are shown as dashed curves. Accurate energies (obtained from experimentally accurate atomic data and dissociation energies) for the complete-dissociation limit and the equilibrium well depth are shown as red and gray horizontal markers. (b) The corrected adiabatic PEC $\mathbf{V}_i^{\mathrm{a-di}}$ is shown in blue PEC, and the dashed curves from panel a are repeated for comparison.

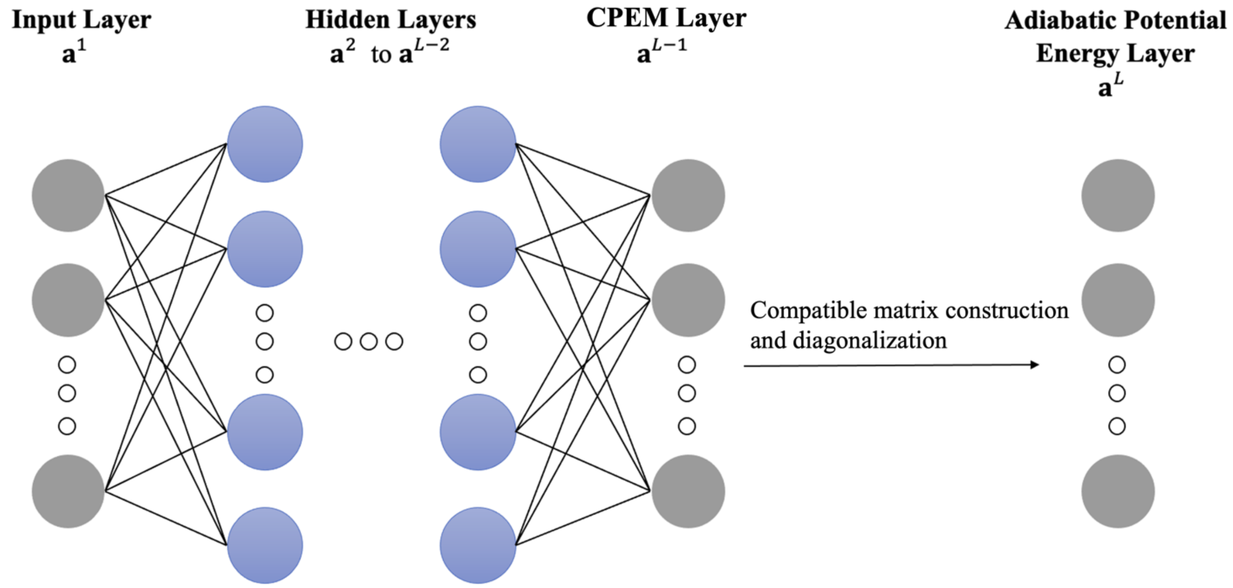

Figure S5. Schematic illustration of the parametrically managed CDNN architecture.

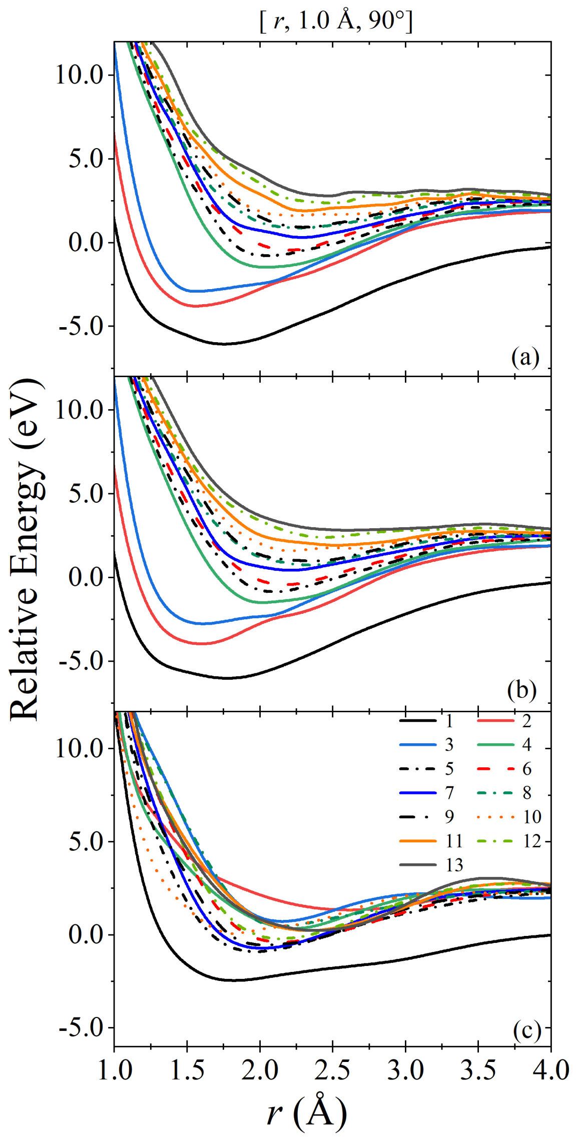

Figure S6. Sample potential surface cut for geometries with $d = 1$ Å and $\gamma = 90°$. (a) Electronic structure calculations. (b) Fitted adiabatic energies. (c) Diagonal elements of compatibility matrix.

$$N_2(X^1\Sigma_g^+, v = 5, j = 80) + O(^1S) \xrightarrow{E_{rel} = 3.0\ \text{eV}} N_2(X^1\Sigma_g^+, v' = 6.0, j' = 34.8) + O(^1D)$$

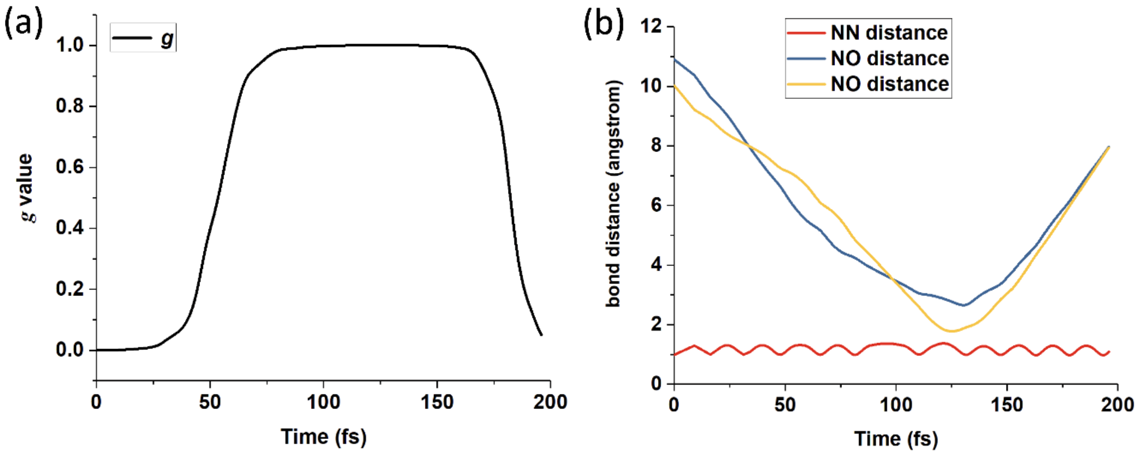

$$N_2(X^1\Sigma_g^+, v = 5, j = 80) + O(^1S) \xrightarrow{E_{rel} = 3.0\ \text{eV}} N_2(A^3\Sigma_u^+, v = 0.0, j = 78.3) + O(^3P)$$

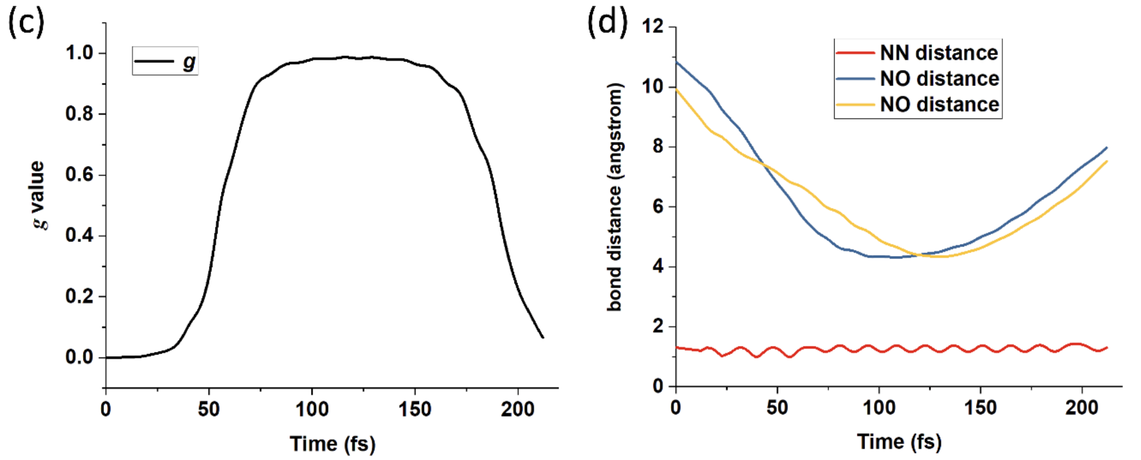

$$N_2(X^1\Sigma_g^+, v = 5, j = 80) + O(^1S) \xrightarrow{E_{rel} = 3.0\ \text{eV}} NO(X\ ^2\Pi_r, v' = 0.0, j' = 37.5) + N(^2D)$$

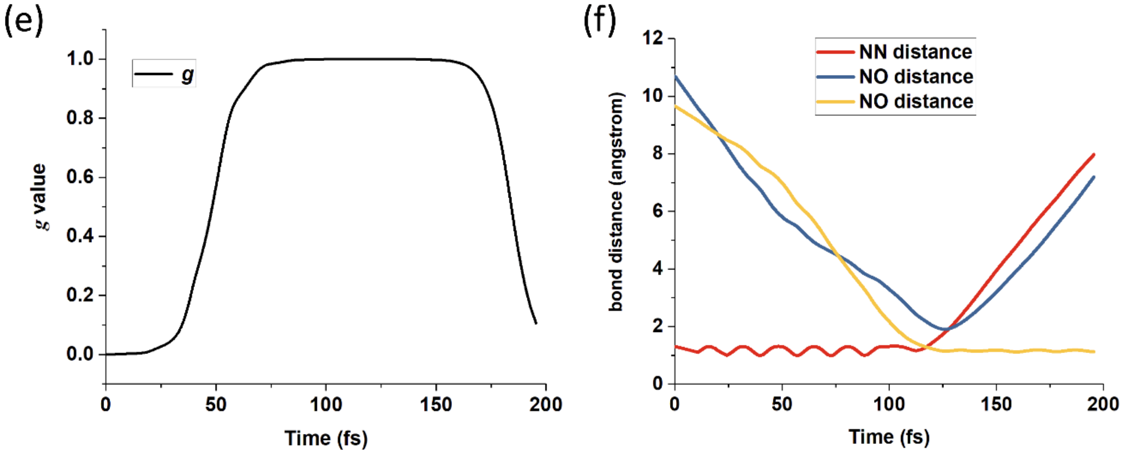

Figure S7. Values of g (a,c,e) and three diatomic distances (b,d,f) as functions of time.

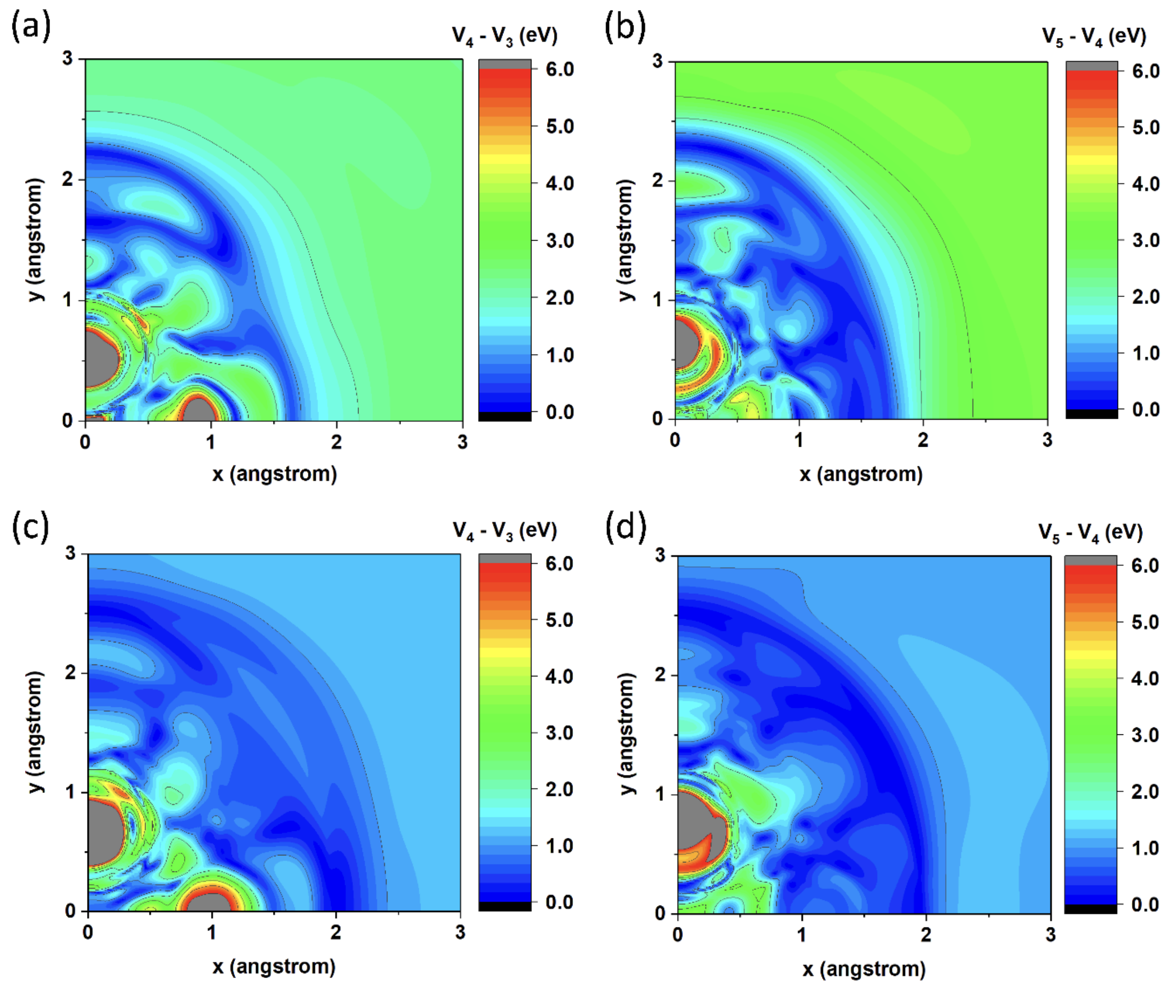

Figure S8. Adiabatic potential energy gap between (a, c) $\alpha = 4$ and $\alpha = 3$, and (b, d) $\alpha = 5$ and $\alpha = 4$ as functions of the position of the O atom. The N_2O is in the *xy* plane. The nitrogen atoms are at $x = 0$, y = ±0.55 Å for panels a and b, and at $x = 0$, y = ±0.70 Å for panels c and d.

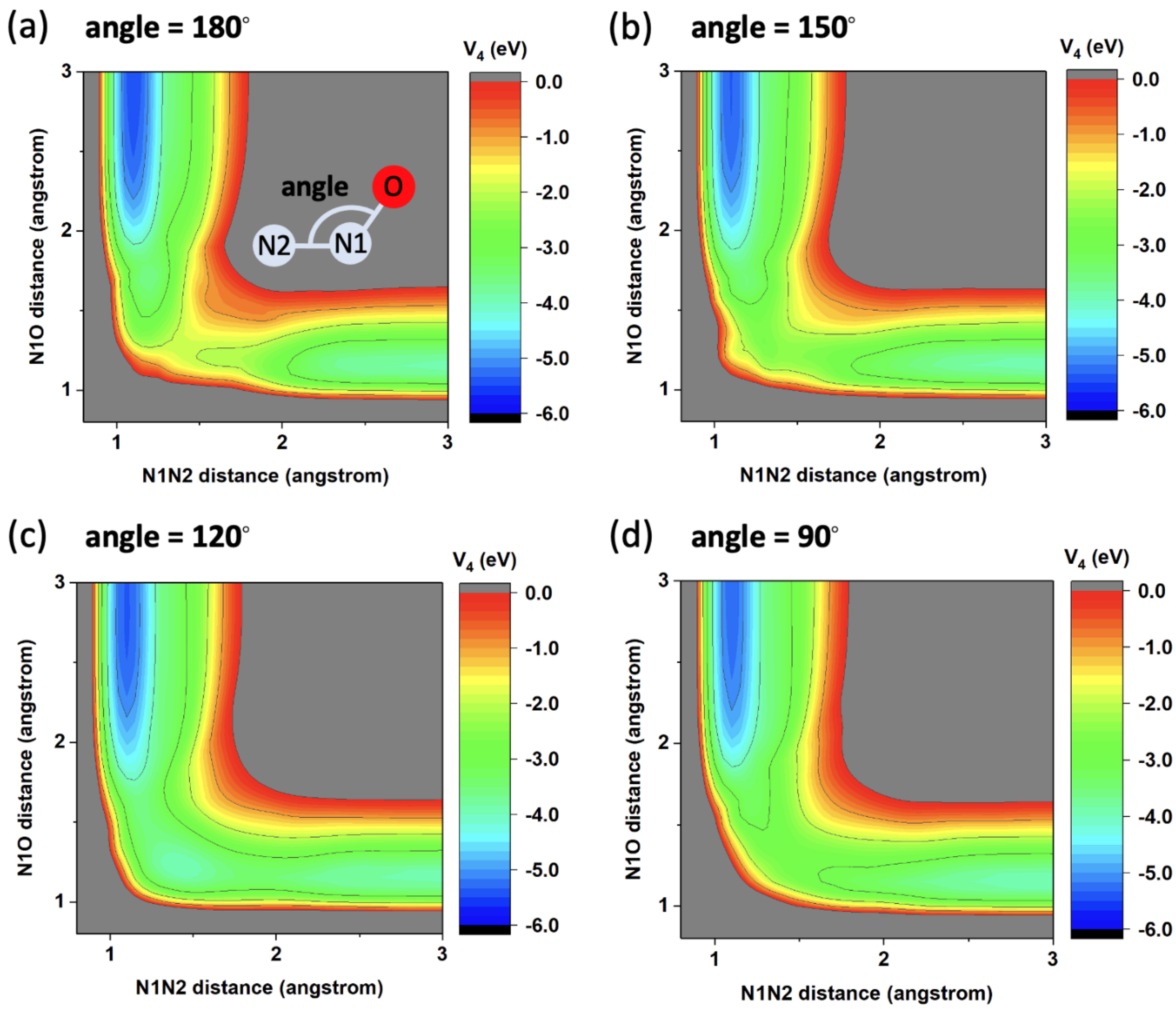

Figure S9. Contour plots of V_4 as a function of N1–O and N1-N2 distance; panels a to d correspond to N2–N1–O angles of 180, 150, 120, and 90 degrees.

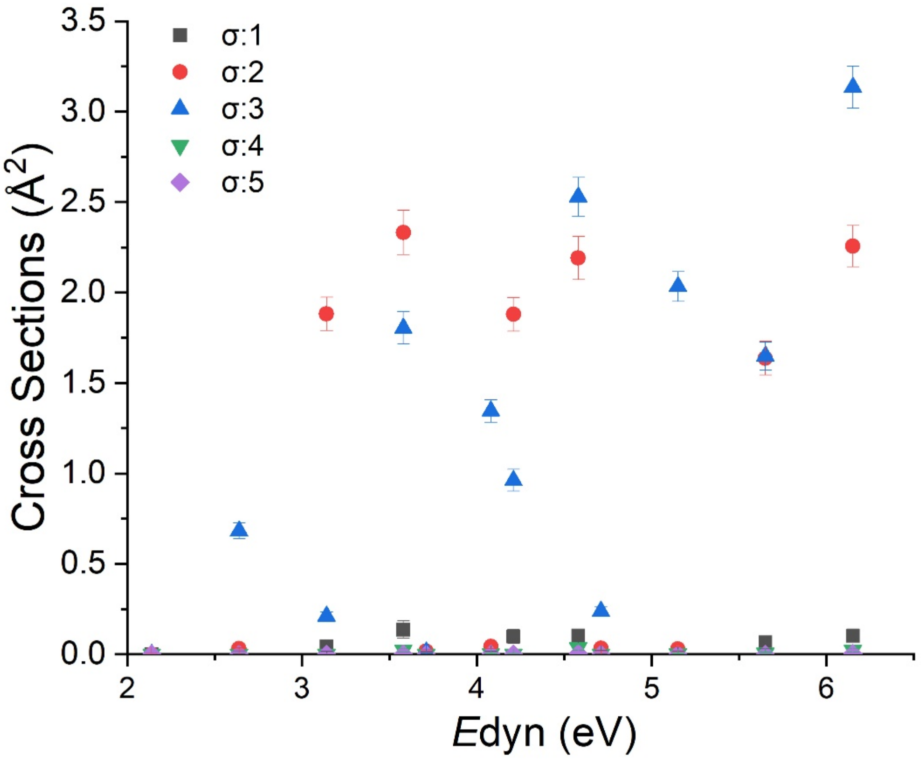

Figure S10. Scatter plot of cross sections with statistical error bars as a function of E_{dyn} for $N_2(X)$ + O(^{1}S) collisions. The five cross sections are defined as follows:

1. Inelastic de-excitation of O to the ^{1}D state: The final state is in arrangement N_2 + O on adiabatic surfaces with α equal to 1, 2, and 3.
2. Inelastic excitation to $N_2(A)$ + O(^{3}P): The final state is in arrangement N_2 + O on adiabatic surface with α equal to 5.
3. Reaction producing NO(X) + N(^{2}D): The final state is in arrangement NO + N on adiabatic surfaces with α equal to 1, 2, 3, 4, and 5.
4. Reaction producing NO(X) + N(^{2}P): The final state is in arrangement NO + N on adiabatic surfaces with α equal to 6, 7, and 8.
5. Reaction producing NO(a) + N(^{4}S): The final state is in arrangement NO + N on adiabatic surface with α equal to 9.

Table S1. The relative energies (ΔE) and the diabatic state order at three asymptotic geometries.

	$N_2(r_{e,NN})$ + O			$NO(r_{e,NO})$ + N			N + N + O		
α [b]	Asymptote	E_{dyn} [c]	δ [d]	Asymptote	E_{dyn} [c]	δ [e]	Asymptote	E_{dyn} [c]	δ [f]
1	$X^1\Sigma_g^+ + {}^1D$	-2.22	4	$X^2\Pi_r + {}^2D$	1.49	6	${}^4S + {}^4S + {}^3P$	5.71	1
2	$X^1\Sigma_g^+ + {}^1D$	-2.22	2	$X^2\Pi_r + {}^2D$	1.49	7	${}^4S + {}^4S + {}^1D$	7.74	2
3	$X^1\Sigma_g^+ + {}^1D$	-2.22	3	$X^2\Pi_r + {}^2D$	1.49	8	${}^4S + {}^4S + {}^1D$	7.74	3
4	$X^1\Sigma_g^+ + {}^1S$	0.00	8	$X^2\Pi_r + {}^2D$	1.49	9	${}^4S + {}^4S + {}^1D$	7.74	4
5	$A^3\Sigma_u^+ + {}^3P$	2.06	1	$X^2\Pi_r + {}^2D$	1.49	5	${}^4S + {}^2D + {}^3P$	8.15	5
6	$B^3\Pi_g + {}^3P$	3.20	6	$X^2\Pi_r + {}^2P$	2.69	3	${}^4S + {}^2D + {}^3P$	8.15	6
7	$B^3\Pi_g + {}^3P$	3.20	7	$X^2\Pi_r + {}^2P$	2.69	11	${}^4S + {}^2D + {}^3P$	8.15	7
8	$B^3\Pi_g + {}^3P$	3.20	5	$X^2\Pi_r + {}^2P$	2.69	12	${}^4S + {}^2D + {}^3P$	8.15	8
9	$W^3\Delta_u + {}^3P$	3.23	11	$a^4\Pi_i + {}^4S$	3.85	1	${}^4S + {}^2D + {}^3P$	8.15	9
10	$W^3\Delta_u + {}^3P$	3.23	12	$b^4\Sigma^- + {}^4S$	5.10	2	${}^4S + {}^2D + {}^3P$	8.15	10
11	$W^3\Delta_u + {}^3P$	3.23	13	$B^2\Pi_r + {}^2D$	7.16	4	${}^4S + {}^2D + {}^3P$	8.15	11
12	$B'^3\Sigma_u^- + {}^3P$	4.03	9	$B^2\Pi_r + {}^2D$	7.07	10	${}^4S + {}^2D + {}^3P$	8.15	12
13	$B'^3\Sigma_u^- + {}^3P$	4.03	10	$B^2\Pi_r + {}^2D$	7.07	13	${}^4S + {}^2D + {}^3P$	8.15	13

[a] The table can be explained by considering the first adiabatic state of $N_2(r_e)$ + O. This state has character of $X^1\Sigma_g^+ + {}^1D$, and its energy is –2.22 eV. This adiabatic electronic state corresponds to diabatic state 4 (and therefore, $V_1 = U_{44}$, where we use the conventions that V_α corresponds to adiabatic potential energy α (numbered from lowest energy to highest energy at any geometry), **U** is the DPEM, and $U_{\delta\delta}$ is a diagonal element of **U**, which diabatic potential energy surface δ.

[b] Adiabatic surface index

[c] E_{dyn} is energy in eV relative to $N_2(X, r_e)$ + O(^{1}S).

[d] $\delta(\alpha, 1, r_{e,1})$

[e] $\delta(\alpha, 2, r_{e,2})$, which is the same as $\delta(\alpha, 3, r_{e,3})$

[f] $\delta(\alpha, 1, r_1 = \infty)$, which is the same as $\delta(\alpha, 2, r_2 = \infty)$ and $\delta(\alpha, 3, r_3 = \infty)$

Table S2. Fitted parameters for the short-range term of ground-state N_2.[a]

Coefficients	Value
$r_{e,\mathrm{N_2}}$	1.098 Å
$D_1^{\mathrm{SR,N_2}}$	224.9157 kcal/mol
a_0	2.7599278840949/Å
a_1	0.2318898277373/Å
a_2	0.1908422945648/Å
a_3	-0.2727504034613/Å
a_4	-0.5345112219335/Å
a_5	1.0857331617073/Å
a_6	1.6339897930305/Å

[a]from ref 2

Table S3. Fitted parameters for $W_{1,\delta}{}^{a}$

δ	1	5,6,7	8	9,10	11,12,13
Character	$N_2(A)$ + O(^{3}P)	$N_2(B)$ + O(^{3}P)	Mixed[b]	Mixed[b]	$N_2(W)$ + O(^{3}P)
a_δ/Å^{-2}	3.3187	19.3171	4.6198	6.6960	9.2448
β_n	0.8887	0.5429	0.8974	0.5026	0.5080
$a_{0,\delta}$/eV	5658083.9534	145721.6205	13801397.4636	15443.4359	16098.9989
$a_{1,\delta}$/eV	-27612545.5363	-15108.2989	-77134570.2939	974.6327	2983.9167
$a_{2,\delta}$/eV	60087849.6434	13756.3574	191858552.6277	2458.0317	3891.1504
$a_{3,\delta}$/eV	-75354670.8070	-364.7871	-273939883.2692	-1933.8374	-1816.4922
$a_{4,\delta}$/eV	58733773.5313	2231.7390	241690690.8885	814.6883	414.5730
$a_{5,\delta}$/eV	-28446736.2084	-1188.2634	-131478520.1160	-409.6383	-275.8663
$a_{6,\delta}$/eV	7943657.1728	40.5559	40801104.4116	181.0276	146.7999
$a_{7,\delta}$/eV	-992807.5071	-6.8829	-5577363.7504	-44.9848	-40.3988

[a] The parameters for δ = 2, 3, and 4 are in Table S1.

[b] These re-ordered curves are complicated by the issue discussed for the adiabatically derived states in Subsection 3.3.1:

The potential energy curve of the δ = 5 state is combined with the ground-state potential energy curve of $N_2(X)$, incorporating an energy shift of 4.13 kcal/mol (the excitation energy of O(^{1}S), at bond distances between 0.6 Å and 1.53 Å. From 1.54 Å to 2.3 Å, the potential is aligned with the 10th state's curve, while for bond lengths beyond 2.3 Å, the energy shifts due to the replacement of N(^{4}S) by N(^{2}D).

A similar treatment is applied to states δ = 12 and 13, which are associated with $N_2(B')$ + O(^{3}P), as these states also do not include N(^{4}S) + N(^{2}P) + O(^{3}P) in the $^1A'$ manifold.

Table S4. Fitted parameters for the short-range term of ground state NO.[a]

Coefficients	Value
$r_{e,\mathrm{dNO}}$	1.1508 Å
a_1	0.896601839 Å
a_2	2.06954271 Å^2
B_{dNO}	-149.47844 kcal/mol
c_1	-0.138534305
c_2	1.889990874
c_3	-4.297653559
c_4	21.43053956
c_5	-44.34782707
c_6	41.07240829
c_7	-10.90996258
c_8	-10.68726082
c_9	9.189735458
c_{10}	-2.201965266

[a]from ref 3

Table S5. Fitted parameters for $W_{2,\delta}$[a]

δ	1	2	3	4	10	11,12,13
Character	NO(a)+N(^{4}S)	NO(b)+N(^{4}S)	Mixed[b]	Mixed[b]	Mixed[b]	Mixed[b]
a_δ/Å^{-2}	7.2232	13.8190	3.6069	3.6196	3.8083	9.1385
β_n	0.7583	0.3973	0.7496	0.7611	0.7418	0.8229
$a_{0,\delta}$/eV	84282.3385	27433.5549	115911.2928	123262.0128	89409.7997	1037945.0954
$a_{1,\delta}$/eV	-172085.5623	9529.7486	-283917.4091	-299719.1066	-191557.1860	-3833076.3354
$a_{2,\delta}$/eV	196632.4138	1966.6217	369598.5084	376445.5442	222796.8573	7301659.5040
$a_{3,\delta}$/eV	-138121.1593	-690.8387	-308780.7952	-298675.7481	-167187.8040	-9036595.7132
$a_{4,\delta}$/eV	64661.9677	139.1330	175671.8621	162037.1866	87308.4877	7727121.2794
$a_{5,\delta}$/eV	-16969.7646	-57.4103	-67964.4415	-61709.1163	-32494.3894	-4477649.4856
$a_{6,\delta}$/eV	2707.8350	25.7407	16658.3095	15493.6486	8061.3534	1605976.7703
$a_{7,\delta}$/eV	-782.2357	-6.2704	-1992.8253	-1883.9882	-993.5595	-270983.1356
ε_δ/Å^{-2}						48.5073
d_δ						5.1098

[a] The parameters for δ = 5, 6, 7, 8, and 9 are in Table S2.

[b] These re-ordered curves are complicated by the issue discussed for the adiabatically derived states in Subsection 3.3.1:

For states δ = 3, 4, and 10, the three-segment approach is used because these states correspond to NO(X) + N(^{2}P), dissociating to N(^{4}S) + N(^{2}P) + O(^{3}P), which is not part of the 13-state $^1A'$ manifold.

Likewise, mixed potential energy curves are applied for the degenerate states δ = 11, 12, and 13. combining the potential energies of NO (B) + N(^{2}D) at shorter bond distances (0.6 Å to 1.5 Å) with the potential of δ = 11 at medium bond distances (1.5 Å to 3.0 Å) and incorporating the energy shift from N(^{2}D) to N(^{4}S). The dissociation of NO(B) + N(^{2}D) leads to N(^{2}D) + N(^{2}D) + O(^{3}P), which is not included in the 13-state $^1A'$ manifold.

Table S6. Mean unsigned errors (MUEs in meV) of the adiabatic energies and the distribution of geometry points (in %) in various energy ranges (E in eV) of the final fit for the thirteen-state $^1A'$ manifold of N_2O.

	<–2		–2 to 3		3 to 13		13 to 33		>33		All
State	%	MUE	%	MUE	%	MUE	%	MUE	%	MUE	MUE
1	43	31	52	21	3	66	1	102	1	124	29
2	34	34	60	24	4	56	1	78	1	87	30
3	29	33	65	25	4	70	1	83	1	97	31
4	26	40	67	28	5	65	1	89	1	108	35
5	19	38	73	30	5	64	1	88	2	112	35
6	12	43	79	35	6	75	1	86	2	96	40
7	9	42	82	36	6	75	2	91	2	78	40
8	7	44	83	38	7	80	2	118	2	103	43
9	1	46	88	41	7	86	2	125	2	114	47
10	0	61	88	47	8	100	2	127	2	116	54
11	0	91	84	49	11	114	2	168	2	171	61
12	0	0	78	55	18	107	3	151	2	158	68
13	0	0	70	69	24	150	4	392	2	274	105
All	14	34	74	37	8	101	2	201	2	133	48

Table S7. Experimental dissociation energy of N_2O

The dissociation energy from the minimum of the potential energy surface of N_2O to the minimum of the potential energy curve of N_2, with O infinitely separated, is called D_e.

The dissociation energy from ground state of N_2O to the ground state of N_2, with O infinitely separated, is called D_e.

	Quantity	Value (eV)	Reference
A	$\Delta H_{f,0}$ (O)	2.5584	B. Ruscic and D. H. Bross, Active Thermochemical Tables (ATcT) values based on ver. 1.202 of the Thermochemical Network (2024); available at ATcT.anl.gov
B	$\Delta H_{f,0}$ (N_2O)	0.8915	B. Ruscic and D. H. Bross, *op. cit.*
C	Excitation energy of $O(^1D)$	1.9674	C. E. Moore, Tables of Spectra of Hydrogen, Carbon, Nitrogen, and Oxygen Atoms and Ions, in *CRC Series in Evaluated Data in Atomic Physics*, edited by J. W. Gallagher (CRC Press, Boca Raton, FL, 1993).
D	ZPE of N_2	0.1458	I. M. Alecu, J. Zheng, Y. Zhao, and D. G. Truhlar, Computational Thermochemistry: Scale Factor Databases and Scale Factors for Vibrational Frequencies Obtained from Electronic Model Chemistries, Journal of Chemical Theory and Computation **6**, 2872-2887 (2010).
E	ZPE of N_2O	0.2936	I. M. Alecu, *et al.*, *op. cit.*
$F = A - B$	$D_0[N_2O \rightarrow N_2 + O(3P)]$	1.67	
$G = F + C$	$D_0[N_2O \rightarrow N_2 + O(^1D)]$	3.63[a]	
$H = G + D - E$	$D_e[N_2O \rightarrow N_2 + O(^1D)]$	3.49	

[a] This value agrees well with the experimental value of 3.65 eV in Table VIII of Selwyn, G. A.; Johnston, H. S. Ultraviolet absorption spectrum of nitrous oxide as a function of temperature and isotopic substitution. J. Chem. Phys. **74**, 3791-3803 (1981).

Table S8. Relative Adiabatic Potential Energies in eV as function of the bond angle of NNO

	Surface	180°	130°	110°
Hopper	1 $^1A'$	0.0	2.1	4.9
	2 $^1A'$	7.2	5.2	8.9
Present	1 $^1A'$	0.0	1.9	4.5
	2 $^1A'$	7.5	5.7	5.2

Table S9. Statistical errors (one standard deviation) of cross sections as functions of initial conditions for $N_2(X)$ + O(^{1}S) collisions

E_{rel}	v	j	$E_{dyn}{}^{b}$	σ:1 O(^{1}D)	σ:2 O(^{3}P)	σ:3 N(^{2}D)	σ:4 N(^{2}P)(	σ:5 N(^{4}S)
2	0	0	2.14	0.00	0.00	0.00	0.00	0.000
2.5	0	0	2.64	0.00	0.01	0.01	0.00	0.000
3	0	0	3.14	0.01	0.02	0.03	0.00	0.000
2	5	0	3.58	0.03	0.09	0.03	0.00	0.000
2	0	80	3.71	0.01	0.00	0.04	0.00	0.000
2.5	5	0	4.08	0.04	0.09	0.06	0.00	0.000
2.5	0	80	4.21	0.01	0.01	0.06	0.00	0.000
3	5	0	4.58	0.03	0.09	0.08	0.00	0.000
3	0	80	4.71	0.01	0.01	0.08	0.00	0.000
2	5	80	5.15	0.05	0.12	0.09	0.01	0.001
2.5	5	80	5.65	0.04	0.12	0.11	0.02	0.002
3	5	80	6.15	0.04	0.11	0.12	0.01	0.004

Table S10. Number of trajectories leading to each outcome as functions of initial conditions for $N_2(X)$ + $O(^1S)$ collisions

E_{rel}	v	j	E_{dyn}^b	σ:1 O(^{1}D)	σ:2 O(^{3}P)	σ:3 N(^{2}D)	σ:4 N(^{2}P)(	σ:5 N(^{4}S)
2	0	0	2.14	0	1	0	0	0
2.5	0	0	2.64	2	7	10	0	0
3	0	0	3.14	5	21	190	0	0
2	5	0	3.58	11	382	167	0	0
2	0	80	3.71	3	5	561	0	0
2.5	5	0	4.08	31	318	664	1	0
2.5	0	80	4.21	4	19	993	2	0
3	5	0	4.58	20	329	1062	5	0
3	0	80	4.71	5	19	1413	2	0
2	5	80	5.15	35	469	1163	12	1
2.5	5	80	5.65	36	426	1564	19	3
3	5	80	6.15	32	397	1872	14	1

Table S11. Number of pointer-state switches for different pairs of states. Note that we did not observe any pointer-state switches involving electronic states beyond state 8. The convention used in the table is that a switch is from an “old” surface to a “new” surface.

old \ new	**1**	**2**	**3**	**4**	**5**	**6**	**7**	**8**	**9**	**10**	**11**	**12**	**13**
**1**				1									
**2**			1	2									
**3**	1	1		7	7								
**4**	82	126	494		2940	58	40	155	99	59	56	12	7
**5**	1	3	4	55		1	1			1	1		
**6**			1	3	2								
**7**				1									

Table S12. The numbers of trajectories with 1, 2, and 3 pointer-state switches for each initial condition for $N_2(X)$ + $O(^1S)$ collisions

E_{rel}	v	j	E_{dyn}[b]	Number of trajectories with 1 switch	with 2 switches	with 3 switches
2	0	0	2.14	1	0	0
2.5	0	0	2.64	12	0	0
3	0	0	3.14	46	0	0
2	5	0	3.58	439	9	1
2	0	80	3.71	60	4	0
2.5	5	0	4.08	466	11	1
2.5	0	80	4.21	105	4	0
3	5	0	4.58	504	7	0
3	0	80	4.71	151	1	1
2	5	80	5.15	737	20	1
2.5	5	80	5.65	741	16	0
3	5	80	6.15	771	15	1
Total[a]				4033	87	5

[a]4033 + 2*87 + 3*5 = 4222.

Table S13. Maximum absolute errors (MaxAE in eV) in the final fit[a]

	State 1	State 2	State 3	State 4	State 5	State 6	State 7	State 8	State 9	State 10	State 11	State 12	State 13
MaxAE	0.42	0.35	0.51	0.86	1.34	1.20	1.27	3.36	3.36	3.20	9.72	8.34	3.88
Adiabatic energy	51.34	51.39	51.46	51.49	51.63	52.06	52.27	52.48	52.83	52.83	53.23	54.61	62.99

[a] The table shows the maximum absolute error in the fit to each state and the associated adiabatic relative energy at that point (both in eV) We see that the largest errors occur where the energy is very high. It is not necessary to fit the relative energies precisely high on the repulsive wall; it is only necessary that the fitted energy be high enough that no trajectory will go there. In fact, the highest errors occur for geometries where N is far away from NO and where NO has a very small bond length of 0.65Å.

Additional supporting information

Additional data is available online with the journal article in a zip file containing 5 separate files.

SI_Meng_x.dat	internuclear distances of N_2O (in Å) for the final fitting data set
SI_Meng_y.dat	adiabatic potential energies of the 13 $^1A'$ states in eV for the final fitting data set
SI_Meng_1Ap_N2O_CASSCF.inp	an example input file for SA-CASSCF calculations of 13 $^1A'$ states for N_2O
SI_Meng_1Ap_N2O_CASPT2.inp	an example input file for CASPT2 calculation of 13 $^1A'$ states for N_2O by reading the CASSCF wave function information file generated by CASSCF calculations
SI_Meng_1Ap_N2O.xyz	the Cartesian coordinate file for 1Ap_N2O_CASSCF.inp
SI_Meng_1Ap_N2O_ANT.inp	an example input file ofdynamics calculations with semiclassical method κCSDM for 13 $^1A'$ states for N_2O

Fortran routines

A set of Fortran routines containing the fit is openly available in the latest version of the *ChemPotPy* repository[11] (https://github.com/shuyinan/chempotpy). These routines can generate the potential surfaces and their gradients in both the compatible and adiabatic representations.

References

[1] Brown F. B.; Truhlar, D. G. A New Semiempirical Method of Correcting Large-Scale Configuration Interaction Calculations for Incomplete Dynamic Correlation of Electrons. *Chem. Phys. Lett.* **1985**, *117*, 307-313.

[2] Varga, Z.; Truhlar, D. G. Potential energy surface for high-energy N+ N_2 collisions. *Phys. Chem. Chem. Phys.* **2021***, 23*, 26273-26284.

[3] Varga, Z.; Liu, Y.; Li, J.; Paukku, Y.; Guo, H.; Truhlar, D. G. Potential energy surfaces for high-energy N + O_2 collisions. *J. Chem. Phys.* **2021**, *154*, no. 084304.

[4] Becke, A. D.; Johnson, E. R. A density-functional model of the dispersion interaction. *J. Chem. Phys.* **2005***, 122*, no. 154101.

[5] Verma, P.; Wang, B.; Fernandez, L. E.; Truhlar, D. G. Physical Molecular Mechanics Method for Damped Dispersion *J. Phys. Chem.* **2017***, 121*, 285-2862.

[6] Grimme, S.; Antony, J.; Ehrlich, S.; Krieg, H. A consistent and accurate ab initio parametrization of density functional dispersion correction (DFT-D) for the 94 elements H-Pu. *J. Chem. Phys.* **2010***, 132*, no. 154104.

[7] Grimme, S.; Ehrlich, S.; Goerigk, L. Effect of the damping function in dispersion corrected density functional theory. *J. Comput. Chem.* **2011***, 32*, 1456−1465.

[8] Hopper, D. G. (1984). Ab initio multiple root optimization MCSCF study of the $C_{\infty v}/C_s$ excitation spectra and potential energy surfaces of N_2O. *J. Chem. Phys.* **1984***, 80*, 4290-4316.

[9] Yu, H. Y. S.; He, X.; Li, S. H. L.; Truhlar, D. G. MN15: A Kohn- Sham global-hybrid exchange-correlation density functional with broad accuracy for multi-reference and single-reference systems and noncovalent interactions. C*hem. Sci.* **2016***, 7*, 5032−5051.

[10] Frisch, M. J.; Pople, J. A.; Binkley, J. S. Self-consistent molecular orbital methods 25. Supplementary functions for Gaussian basis sets. *J. Chem. Phys.* **1984***, 80*, 3265−3269.

[11] Shu, Y.; Varga, Z.; Zhang, D.; Truhlar, D. G. ChemPotPy: A Python Library for Analytic Representations of Potential Energy Surfaces and Diabatic Potential Energy Matrices. *J. Phys. Chem. A* **2023**, *127*, 9635-9640.
